# Dual roles for a tick protein disulfide isomerase during the life cycle of the Lyme disease agent

**DOI:** 10.1128/mbio.01754-24

**Published:** 2024-10-29

**Authors:** Xiaotian Tang, Yingjun Cui, Ushuu Namarra, Xiuqi Tian, Freddie Rivas-Giorgi, Erol Fikrig

**Affiliations:** 1 Zhejiang Key Laboratory of Biology and Ecological Regulation of Crop Pathogens and Insects, Institute of Insect Sciences, College of Agriculture and Biotechnology, Zhejiang University, Hangzhou, China; 2 Section of Infectious Diseases, Department of Internal Medicine, School of Medicine, Yale University, New Haven, Connecticut, USA; 3 History of Science, Medicine, and Public Health Program, Yale College, New Haven, Connecticut, USA; 4 Molecular Biochemistry and Biophysics Program, Yale College, New Haven, Connecticut, USA; McGovern Medical School, Houston, Texas, USA

**Keywords:** protein disulfide isomerase, *Ixodes scapularis*, Lyme disease, *Borrelia burgdorferi*, infection, tick

## Abstract

The protein disulfide isomerase (PDI) family is a group of enzymes that have thiol-disulfide oxidoreductase, disulfide isomerase, and redox-dependent chaperone activities. PDIs facilitate diverse infections in mammalian hosts by directly binding to pathogens, immunomodulation, or enabling microbial invasion of host cells. PDI homologs within pathogens are also potential virulence factors. However, whether PDIs within blood-feeding ticks influence microbial infection remains unknown. In this study, we investigated the role of *Ixodes scapularis* PDIs, on the Lyme disease agent, *Borrelia burgdorferi. I. scapularis* has five PDIs (IsPDIs), and *IsPDIA6* gene expression is reduced upon *B. burgdorferi* infection in the tick. IsPDIA6-mediated trypsin inhibitor gene expression contributes to *B. burgdorferi* colonization within the tick midgut. IsPDIA6 is also secreted into the host during tick feeding, alters cytokine/chemokine expression at the tick bite site, and influences the initial stage of bacterial infection in mice. These data demonstrate that a PDI from a blood-feeding vector plays a role in the life cycle of an extracellular pathogen.

**IMPORTANCE:**

Vector-borne diseases are a leading cause of death and illness worldwide, and more than 80% of the global population live in areas at risk from at least one major vector-borne disease. In this study, we demonstrate a dual role of a specific *Ixodes* tick protein disulfide isomerase (PDI) in inhibiting the ability of the Lyme disease agent to colonize ticks and also in enhancing the initial stage of spirochete infection of mice. This study represents a novel conceptual advancement that a PDI from a blood-feeding vector plays important roles in the life cycle of an extracellular pathogen.

## INTRODUCTION

Protein disulfide isomerases (PDIs) belong to the thioredoxin superfamily of redox proteins (1) and act as both a thiol-disulfide oxidoreductase and a molecular chaperone in many organisms (1, 2). The typical PDI protein contains a signal peptide, two thioredoxin-like catalytic domains (a and a′) with two active site motifs (Cys-Gly-His-Cys, CGHC), two non-catalytic domains (b and b′), and an endoplasmic reticulum (ER) retention signal (3). The predominant function of PDIs is preserving the native conformation and stability of other proteins via the formation, isomerization, and rearrangement of disulfide (S-S) bonds in the ER (4).

Albeit primarily localized in the ER, PDIs are also found in the nucleus, cytosol, cell surface, and extracellular space (1). This reflects that PDIs may have specialized functions, and their roles are not limited within the ER. Indeed, PDIs and PDI-mediated pathways influence the infection of intracellular pathogens, including viral, bacterial, and parasitic infections (5–8). PDIs influence microbes in mammalian hosts via cell surface disulfide reductases that enable pathogens to invade host cells, directly binding to pathogens or immunomodulating the environment. For instance, cell invasion by *Anaplasma phagocytophilum* and *Ehrlichia chaffeensis* is facilitated by host PDIs, and the PDI-mediated cell invasion is impeded by a PDI-inhibiting membrane-impermeable antibiotic mix, bacitracin (5, 6). In addition, *Leishmania*, *Trypanosoma*, and *Plasmodium* have their own PDIs that act as important virulence factors (9–11).

Vector-borne diseases result from infections transmitted to humans and other animals by blood-feeding arthropods. These diseases pose significant threats to public health, leading to a huge burden of morbidity and mortality worldwide. Knowledge about whether vector PDIs influence pathogens infectivity is largely limited (12); moreover, further investigation is needed to determine whether PDIs or PDIs-mediated pathways can influence infection by extracellular microbes. *Borrelia burgdorferi* is a gram-negative extracellular spirochete bacterium that causes Lyme disease. *B. burgdorferi* is mainly transmitted by black-legged ticks (*Ixodes scapularis*) in North America (13). The Centers for Disease Control and Prevention reports that over 476,000 people in the U.S. are diagnosed and treated for Lyme disease, making it the most common tick-borne pathogen in North America (14). Lyme disease can usually be treated with a few weeks of antibiotic therapy. However, if left untreated, infection can lead to serious complications (15). Our previous study demonstrated that a tick protein disulfide isomerase A3 (PDIA3) was associated with *B. burgdorferi* acquisition (12). In this study, we explored the importance of various *I. scapularis* PDIs in *B. burgdorferi* acquisition, colonization, and transmission. We demonstrated the dual role of a specific tick protein disulfide isomerase in inhibiting *B. burgdorferi* colonization of *I. scapularis* and in enhancing the initial stage of spirochete infection of mice. The data generated here will provide novel targets for targeting *B. burgdorferi* and may be applicable to other arthropods and vector-borne infections.

## RESULTS

### Characterization of *I. scapularis* protein disulfide isomerases

**Fig 1:**
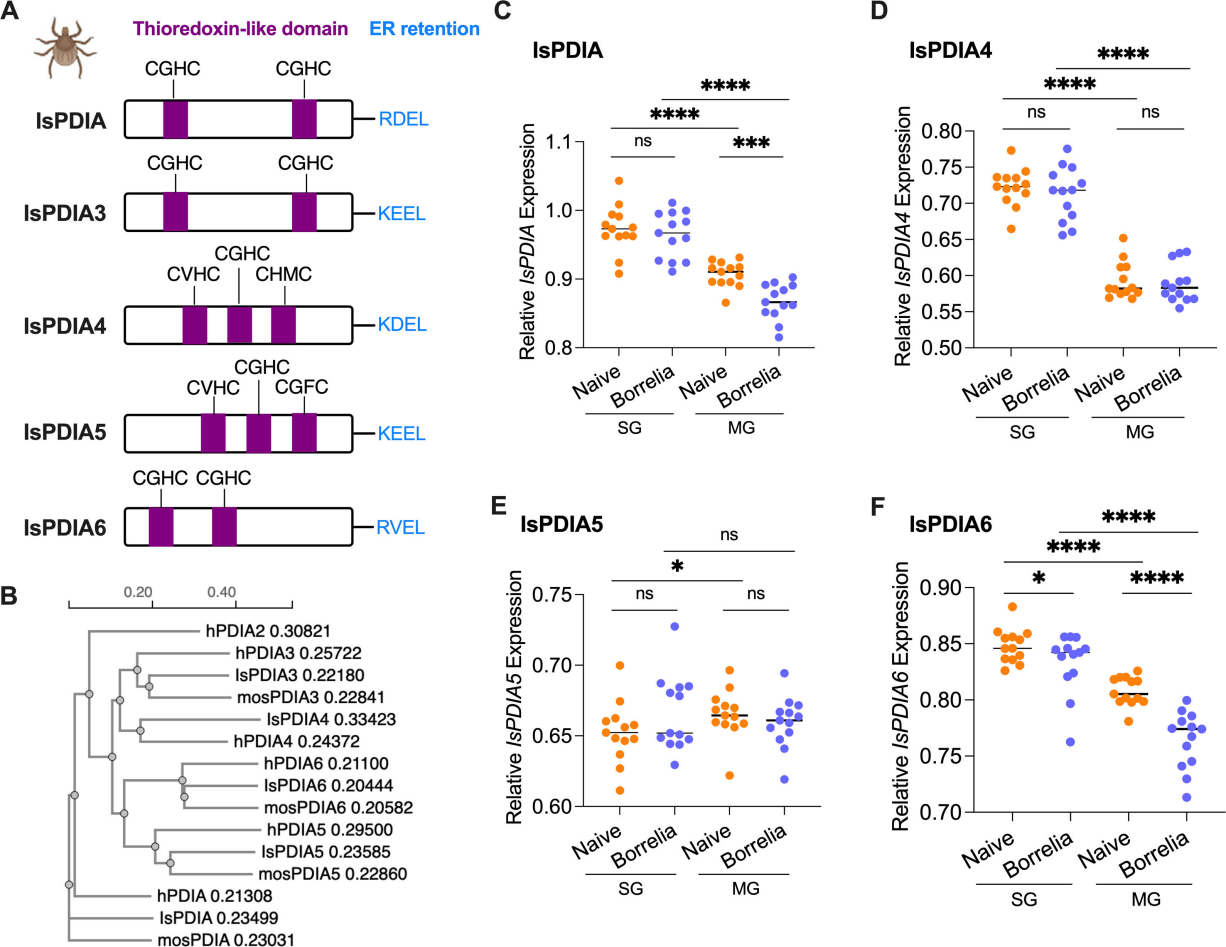
Protein domain analysis and gene expression of *IsPDIAs* in ticks. (**A**) Domain organization of five IsPDIA proteins. The catalytically active domains are represented in violet with active sites (CXXC) noted. C-terminal ER retention sequences are represented in light blue with their amino acid composition denoted. (**B**) Simplified phylogenetic tree diagram showing the relationships among tick IsPDIAs, human PDIAs (hPDIA), and mosquito *A. aegypti* PDIAs (mosPDIA). The numbers behind gene names are branch length. (**C**) Gene expression of *IsPDIA* in tick salivary glands (SG) and midgut (MG), without or upon *B. burgdorferi* infection. The tick *actin* gene was used as a reference gene for all qPCR analysis. (**D**) Gene expression of *IsPDIA4* in tick salivary glands and midgut, without or upon *B. burgdorferi* infection. (**E**) Gene expression of *IsPDIA5* in tick salivary glands and midgut, without or upon *B. burgdorferi* infection. (**F**) Gene expression of *IsPDIA6* in tick salivary glands and midgut, without or upon *B. burgdorferi* infection. For all the data, each dot represents one biological replicate. Statistical significance was assessed using a non-parametric Mann-Whitney test (*P < 0.05; ***P < 0.001; ****P < 0.0001; ns, *P* > 0.05).

A BlastP search in NCBI (https://www.ncbi.nlm.nih.gov/) and VectorBase (https://vectorbase.org/vectorbase/) with six human PDIAs (PDIA1-6) and mosquito (*Aedes aegypti*) PDIAs identified five PDIA homologs in the *I. scapularis* genome (16): IsPDIA (ISCP_014339), IsPDIA3 (ISCP_015586), IsPDIA4 (ISCP_034856), IsPDIA5 (ISCP_003869), and IsPDIA6 (ISCP_006970). The PDIA2 homolog is missing in *I. scapularis*. The five identified IsPDIAs have a signal peptide and an endoplasmic retention signal in their carboxy terminus (RDEL, KEEL, KDEL, and RVEL) (Fig. 1A). In addition, all five IsPDIAs have typical thioredoxin-like domains with active sites (CXXC). IsPDIA, IsPDIA3, and IsPDIA6 have two thioredoxin-like catalytic domains with two active site motifs (CGHC), while there are three thioredoxin-like catalytic domains with distinct active site motifs (CVHC, CGHC, CHMC, and CGFC) in IsPDIA4 and IsPDIA5 (Fig. 1A). All these characteristics are consistent with human PDIAs. Phylogenetic tree analysis based on the tick, human, and mosquito protein sequences confirmed each PDIA category (Fig. 1B). IsPDIA3 and IsPDIA4 have the most similarity, and IsPDIA5 and IsPDIA6 are more related to each other than to other IsPDIAs (Fig. 1B ; Fig. S1).

### *IsPDIAs* are highly expressed in *I. scapularis* salivary glands and midguts

To investigate the potential function of IsPDIAs in *I. scapularis*, we examined the gene expression of five identified *IsPDIAs* in nymphal tick salivary glands and midguts. *IsPDIA3* is known to be expressed more abundantly in the tick midgut than salivary gland (12). In this study, we found that the tick salivary glands express significantly higher levels of *IsPDIA*, *IsPDIA4*, and *IsPDIA6* compared to midguts (*P* < 0.0001). In contrast, *IsPDIA5* is expressed more abundantly in tick midguts compared to salivary glands (*P* < 0.05) (Fig. 1C through F). These differential expression patterns suggest that IsPDIAs may exhibit distinct functional roles in tick salivary glands and midguts.

### *IsPDIA* and *IsPDIA6* gene expression is reduced in ticks infected with *B. burgdorferi*

*IsPDIA3* expression is significantly induced in ticks upon *B. burgdorferi* infection (12). We therefore further examined whether the expression of the newly identified *IsPDIA* genes was altered by *B. burgdorferi*. Nymphal ticks were allowed to engorge on either uninfected or *B. burgdorferi*-infected mice. We found that *IsPDIA4* and *IsPDIA5* gene expression was not changed by *B. burgdorferi* (*P* > 0.05) (Fig. 1D and E); however, *IsPDIA* gene expression was significantly downregulated in tick midgut (*P* < 0.001) (Fig. 1C), and *IsPDIA6* gene expression was significantly downregulated in both the tick salivary gland and midgut upon *B. burgdorferi* infection (*P* < 0.05) (Fig. 1F). These data suggest that *IsPDIA* and *IsPDIA6* may have roles during *B. burgdorferi* colonization of the tick midgut and/or transmission from the tick salivary gland.

### *IsPDIA6* impairs *B. burgdorferi* colonization and enhances *B. burgdorferi* transmission

**Fig 2:**
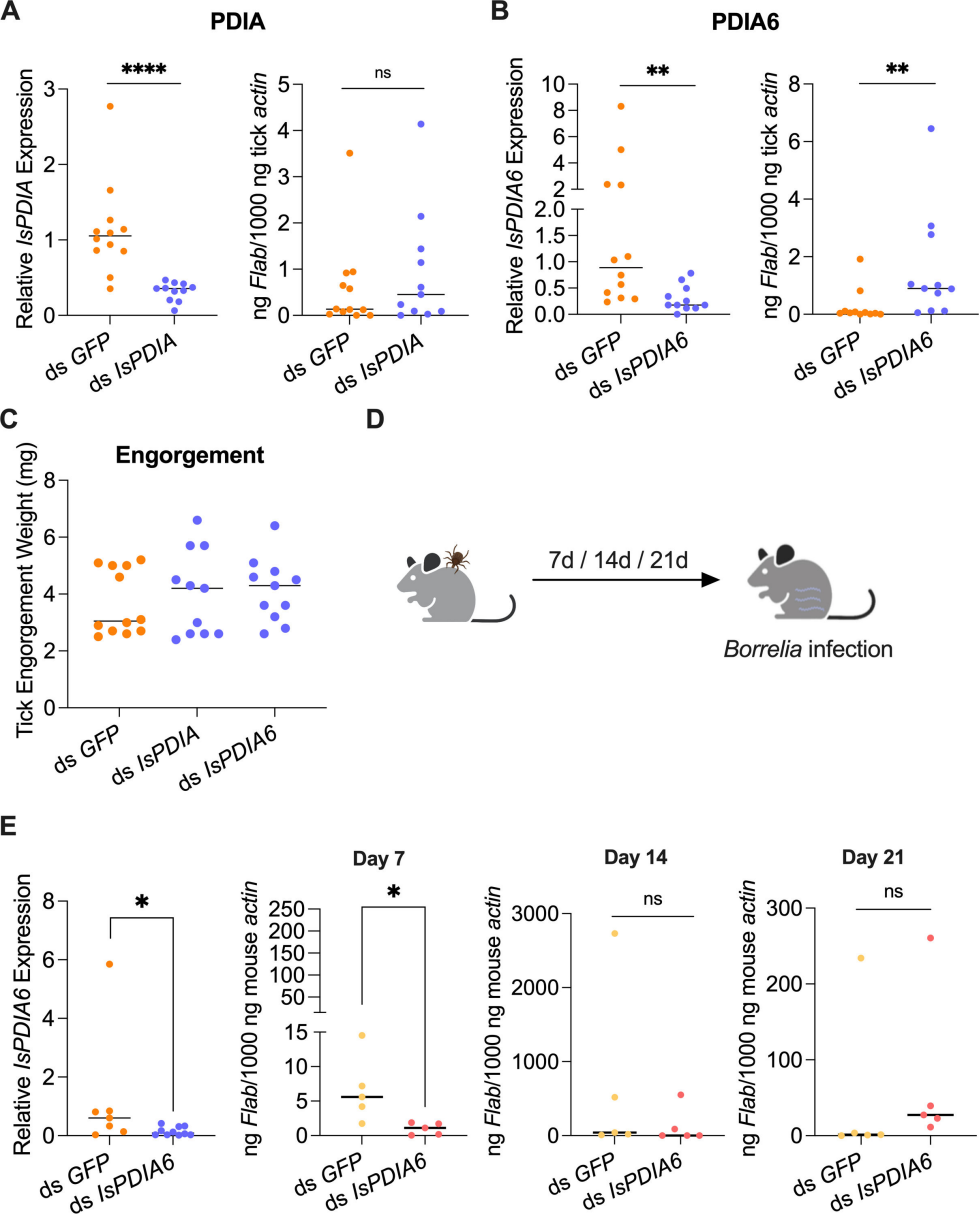
Effects of silencing *IsPDIA* and *IsPDIA6* on *B. burgdorferi* infection. (**A**) Silencing of *IsPDIA* has no effect on the *B. burgdorferi* burden in nymphal tick guts. (**B**) Silencing of *IsPDIA6* significantly increases the *B. burgdorferi* burden in nymphal tick guts. (**C**) Nymphal engorgement weights in *IsPDIA*-, *IsPDIA6*-silenced, and *GFP*-injected nymphs. (**D**) *B. burgdorferi*-infected nymphs microinjected with ds *IsPDIA6* or ds *GFP* were fed on clean mice to assess transmission of the spirochete. (**E**) Silencing of the tick *IsPDIA6* gene significantly decreases the *B. burgdorferi* burden in murine ear tissue at 7 days following the bite of infected ticks. For all the data, each dot represents one biological replicate. Statistical significance was assessed using a non-parametric Mann-Whitney test (*P < 0.05; **P < 0.01; ****P < 0.0001; ns, *P* > 0.05).

Since *IsPDIA* and *IsPDIA6* gene expression in the tick midgut was altered by *B. burgdorferi* infection, we silenced these two genes to assess whether IsPDIA or IsPDIA6 is involved in *B. burgdorferi* acquisition or colonization. We delivered *GFP* (control), *IsPDIA*, or *IsPDIA6* dsRNA into the guts of pathogen-free nymphs by anal pore injection. Then, the silenced ticks were allowed to feed on *B. burgdorferi*-infected mice. Interestingly, we found that the knockdown of *IsPDIA6* significantly increased the *B. burgdorferi* burden in the tick gut (*P* < 0.01), while there was no change in *B. burgdorferi* infection of gut after silencing *IsPDIA* (*P* > 0.05) (Fig. 2A and B). We also silenced *IsPDIA4*, whose expression was not influenced by *B. burgdorferi* infection, and, indeed, we did not observe a significant change in the *B. burgdorferi* burden in the tick gut (Fig. S2). The engorgement weights of ds *IsPDIA6*-injected nymphs and control ds *GFP*-injected nymphs were comparable (*P* > 0.05) (Fig. 2C), suggesting that any influence of *IsPDIA6* on *B. burgdorferi* infection is independent of the ability of the tick to take a blood meal.

Since *IsPDIA6* expression in the tick salivary gland is also altered by *B. burgdorferi* infection, we further examined whether IsPDIA6 has a role in influencing *B. burgdorferi* transmission from the vector to vertebrate host. We silenced *IsPDIA6* gene expression in the salivary gland of infected nymphs by RNA interference and determined whether ticks lacking *IsPDIA6* transmit the Lyme disease agent to mice (Fig. 2D). The data showed a significant decrease of *IsPDIA6* expression in the salivary glands of ds *IsPDIA6*-injected ticks when compared to that in control ds *GFP*-injected ticks (*P* < 0.05) (Fig. 2E), indicating that the knockdown was also successful in *B. burgdorferi-*infected ticks. The *B. burgdorferi* burden in murine skin punches was then assessed by qPCR at 7, 14, and 21 days post tick detachment (Fig. 2D). We found that the mice engorged upon by *IsPDIA6*-knockdown-infected nymphal ticks had a significantly lower *B. burgdorferi* burden in ear tissue at 7 days after infection than the control group (*P* < 0.05) (Fig. 2E). At the later time points, 14 and 21 days, infection levels were unchanged (*P* > 0.05), suggesting that IsPDIA6 affects the establishment of early *B. burgdorferi* infection in the host but does not alter long-term infection. Taken together, among the four tested IsPDIAs, only IsPDIA6 has effects on *B. burgdorferi* acquisition and transmission. We therefore focused on IsPDIA6 for additional studies.

### *IsPDIA6* protein production by *I. scapularis* and IsPDIA6 antibody generation following tick bites

**Fig 3:**
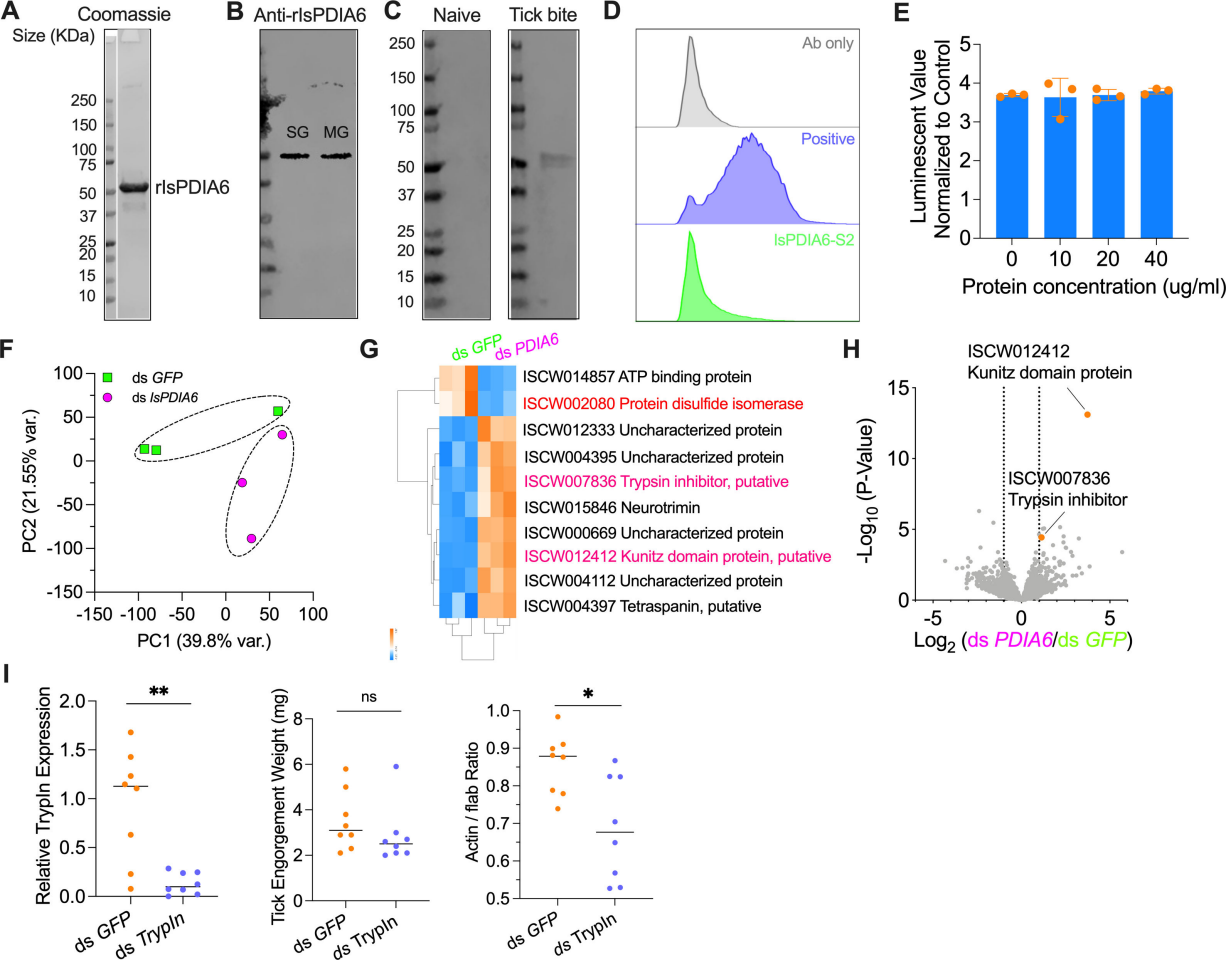
IsPDIA6 protein expression and potential mechanisms of IsPDIA6 on *B. burgdorferi* infection. (**A**) Generation of recombinant IsPDIA6 from a *Drosophila* expression system. The recombinant protein was further analyzed by SDS-PAGE gel with Coomassie Blue staining. (**B**) IsPDIA6 protein is highly expressed in unfed tick salivary glands (SG) and midgut (MG). (**C**) IsPDIA6 antibodies are elicited by natural tick bites. Western blots of recombinant IsPDIA6 by probing with the naïve serum from guinea pigs or serum from guinea pigs bitten by ticks. (**D**) No interaction of rIsPDIA6 with *B. burgdorferi* was identified, as analyzed by flow cytometry. rIsPDIA5 was used as positive control (unpublished data). The background of Alexa Fluor 488-His antibody alone with *B. burgdorferi* is shown in gray. (**E**) rIsPDIA6 has no effect on *B. burgdorferi* viability as determined by BacTiter-Glo assay. The BacTiter-Glo reagent alone was used as control. (**F**) Principal component analysis of transcriptome data from *GFP*-injected and *IsPDIA6*-silenced ticks. Three biological replicates were included in each treatment. (**G**) Hierarchical clustering of differentially expressed genes was generated between *GFP*-injected and *IsPDIA6*-silenced ticks. (**H**) Volcano plot of differentially expressed genes between *GFP*-injected and *IsPDIA6*-silenced ticks. The genes were highlighted by orange color. The gene names can be found in Table S1. (**I**) qPCR assessment of trypsin inhibitor (*TrypIn*) transcript level, nymphal engorgement weights, and qPCR assessment of *B. burgdorferi* flaB levels in guts following RNAi silencing of trypsin inhibitor after feeding on *B. burgdorferi*-infected mice. For all the data, each dot represents one biological replicate. Statistical significance was assessed using a non-parametric Mann-Whitney test (*P < 0.05; **P < 0.01; ns, *P* > 0.05).

We examined the production of IsPDIA6 protein in ticks. We first purified recombinant IsPDIA6 protein (rIsPDIA6) from *Drosophila* S2 insect cells (Fig. 3A). High-titer polyclonal antibodies against rIsPDIA6 were produced by immunizing a group of mice (Fig. S3). We used the murine anti-IsPDIA6 sera to probe unfed nymphal tick salivary gland and midgut total protein, respectively. As expected, IsPDIA6 protein is highly expressed in the tick salivary gland and midgut (Fig. 3B). We then quantified IsPDIA6 protein in the fed tick salivary gland and midgut. The amount of IsPDIA6 per tick was estimated to be approximately 0.2 and 0.03 µg in 100 µg salivary gland and midgut protein, respectively (Fig. S4). We then examined the development of IsPDIA6 antibodies in the animals following naïve tick bite. We assessed the sera from guinea pigs bitten by 30 nymphal ticks (17) and found animals recognized rIsPDIA6 after tick infestation (Fig. 3C). This suggests that IsPDIA6 is secreted out of the tick salivary gland, and IsPDIA6 antibodies are elicited by tick bites.

### *IsPDIA6* does not associate with *B. burgdorferi* and does not alter *B. burgdorferi* viability

As diverse pathogens are associated with mammalian PDIAs during infection (5, 6, 18), we examined whether IsPDIA6 directly interacts with *B. burgdorferi* to affect its infection process. Flow cytometry assay showed that rIsPDIA6 does not directly bind to *B. burgdorferi* (Fig. 3D). We further examined whether IsPDIA6 exhibits borreliacidal activity by incubating *B. burgdorferi* with rIsPDIA6 for 48 h. rIsPDIA6 does not change *B. burgdorferi* viability as assessed by the BacTiter-Glo Microbial Cell Viability Assay (Fig. 3E). These results suggest that IsPDIA6 has no direct effects on *B. burgdorferi* but could still influence *B. burgdorferi* infection by altering host or tick responses.

### *IsPDIA6* influences tick Kunitz domain protein and trypsin inhibitor gene expression

Next, to determine whether the influences of IsPDIA6 on *B. burgdorferi* colonization is the result of physiological responses, we performed RNA-sequencing (RNA-seq) to compare the transcriptomes of control (GFP) and *IsPDIA6*-silenced ticks. Principal component analysis (PCA) and cluster dendrograms revealed that *IsPDIA6*-silenced tick samples formed a separate cluster from the control (*ds* GFP) samples (Fig. 3F and G). In total, 10 genes were significantly differentially expressed at a strict level (*P* < 0.05, FDR < 0.05, and fold change ≥2) in the guts of ds *IsPDIA6*-injected nymphal ticks when compared to that in control ds *GFP*-injected ticks (Fig. 3G; Table S1). As expected, the *IsPDIA6* gene was successfully silenced by RNAi as demonstrated by transcriptome analysis and qPCR validation (Fig. 3G). The gene encoding ATP-binding protein (ISCW014857) was also downregulated after silencing *IsPDIA6*. However, the gene encoding Kunitz domain protein (ISCW012412) was highly upregulated (fold change = 13.3187) (Fig. 3G and H). The other seven upregulated genes include neurotrimin (ISCW015846), trypsin inhibitor (ISCW007836), tetraspanin (ISCW004397), and four uncharacterized proteins (ISCW004112, ISCW000669, ISCW004395, and ISCW012333) (Fig. 3G). We were mostly interested in the Kunitz domain protein and trypsin inhibitor genes because they are both cysteine-rich serine protease inhibitors. Because proteases have the potential to digest *B. burgdorferi* membrane components (19), it is possible that the upregulation of the tick Kunitz domain protein and trypsin inhibitor could protect *B. burgdorferi* from proteases degradation, which would be associated with the increased bacterial burden in the tick gut. The kunitz domain protein gene is short (279 bp) and therefore difficult to silence. We silenced the trypsin inhibitor gene and assessed whether it is related to *B. burgdorferi* survival in ticks. After silencing the trypsin inhibitor gene, we found *B. burgdorferi* burden was significantly decreased in ticks, compared to the controls (*P* < 0.05) (Fig. 3I).

### *IsPDIA6* modulates local host immune responses

**Fig 4:**
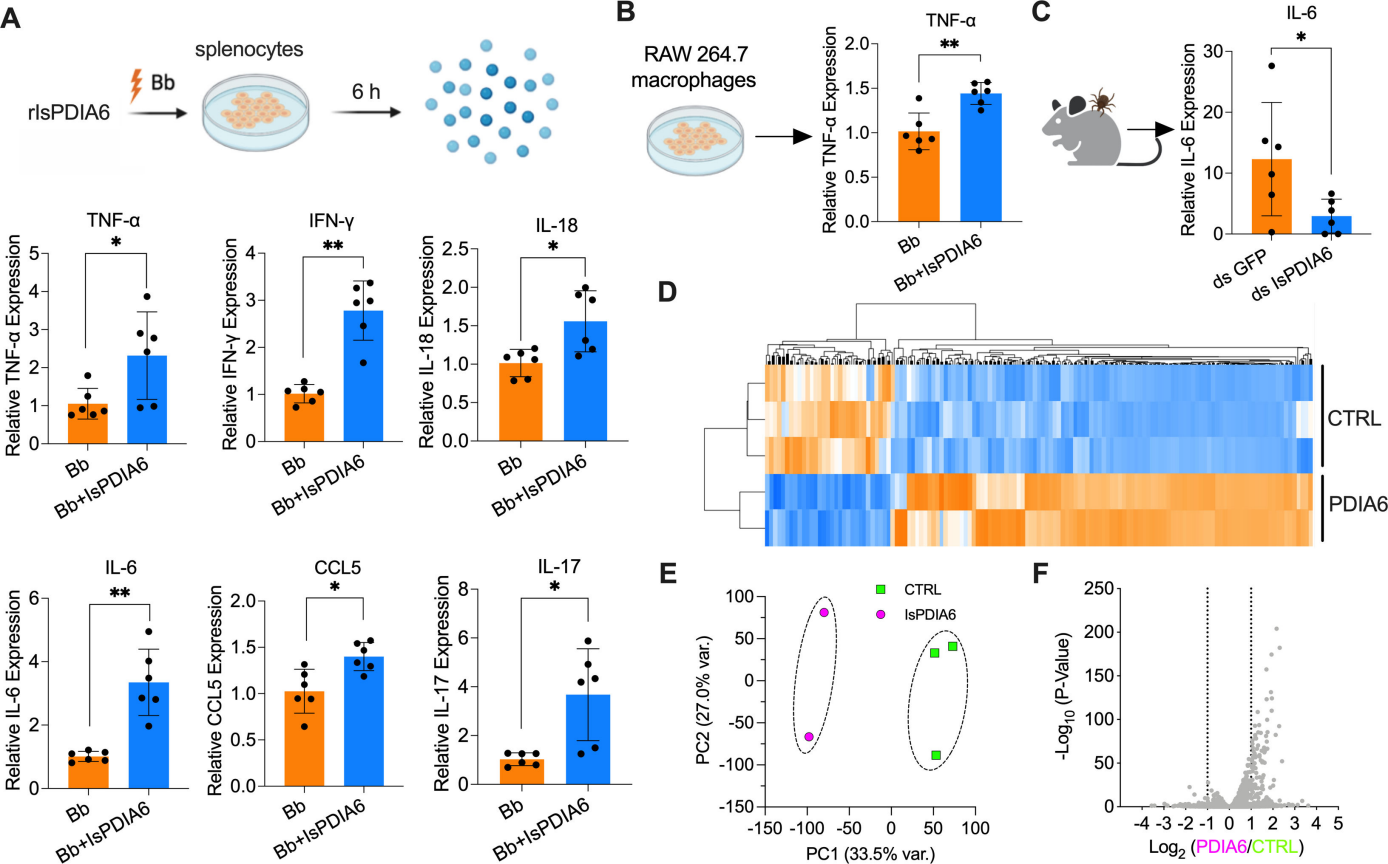
IsPDIA6 modulates immune responses. (**A**) Isolated splenocytes were incubated with *B. burgdorferi* (1 × 10^6^ cells/mL) alone or mixture with rIsPDIA6-S2 for 6 h. Cytokine gene expression was then evaluated by qPCR. rIsPDIA6 significantly induced TNF-α, IFN-γ, IL-18, IL-6, CCL5, and IL-17 gene expression in splenocytes upon *B. burgdorferi* stimulation. (**B**) RAW 264.7 macrophages were incubated with *B. burgdorferi* (1 × 10^6^ cells/mL) alone or mixture with rIsPDIA6-S2 for 6 h. Cytokine gene expression was then evaluated by qPCR. rIsPDIA6 significantly induced TNF-α gene expression in RAW 264.7 macrophages upon *B. burgdorferi* stimulation. (**C**) *B. burgdorferi*-infected nymphs microinjected with ds *IsPDIA6* or ds *GFP* were fed on clean mice for 72 h to assess cytokine genes expression at the tick bite site. IL-6 gene expression was significantly upregulated in the presence of tick IsPDIA6. (**D**) Cluster dendrogram and heatmap of transcriptome data from splenocytes stimulated by *B. burgdorferi* with or without IsPDIA6. Each column represents biological replicates. (**E**) Principal component analysis of transcriptome data from splenocytes stimulated by *B. burgdorferi* with or without IsPDIA6. (**F**) Volcano plot of differentially expressed genes. The gene names can be found in Table S2. For all the data, each dot represents one biological replicate. Statistical significance was assessed using a non-parametric Mann-Whitney test (*P < 0.05; **P < 0.01).

As IsPDIA6 is also secreted into saliva and IsPDIA6 antibodies are elicited by natural tick bites, we examined whether IsPDIA6 changes the mammalian inflammatory microenvironment at the tick bite site during feeding. Indeed, mammalian PDIAs are involved in diverse immunomodulatory activities (20–22). We therefore investigated whether IsPDIA6 could alter host immune responses, potentially influencing immune-pathway-related gene expression as some other tick salivary proteins have been noted to do (23, 24). We isolated murine splenocytes, which consist of a variety of immune cell populations (e.g., T lymphocytes and macrophages), and stimulated the splenocytes using *B. burgdorferi,* with or without rIsPDIA6 for 6 h (Fig. 4A). The expression of cytokine and chemokine genes, which were listed in our previous studies (12, 23), was examined. rIsPDIA6 significantly induced TNF-α, IFN-γ, IL-18, IL-6, CCL5, and IL-17 gene expression in splenocytes upon *B. burgdorferi* stimulation (*P* < 0.05) (Fig. 4A; Fig. S5). As mammalian PDIA inhibitors suppress LPS-mediated proinflammatory cytokines secretion, including TNF-α, IL-1β, and IL-6 (20, 21), we also examine whether rIsPDIA6 alters cytokine and chemokine gene expression in a macrophage cell line. We found that rIsPDIA6 could still induce TNF-α gene expression upon *B. burgdorferi* stimulation (*P* < 0.01) (Fig. 4B; Fig. S6). We further examined whether IsPDIA6 affects host immune responses at the bite site *in vivo* by silencing *IsPDIA6. B. burgdorferi*-infected nymphs were injected with *GFP* or *IsPDIA6* dsRNA and fed on mice for 72 h to assess cytokine gene expression at the bite site. RT-qPCR analysis revealed that the transcription of IL-6 was significantly upregulated in the presence of IsPDIA6 (Fig. 4C).

To further understand the global immunomodulatory roles of IsPDIA6, we utilized RNA-seq to investigate the effect of IsPDIA6 on the murine transcriptome. We compared the transcriptome of splenocytes stimulated by *B. burgdorferi*, with or without IsPDIA6. PCA and cluster dendrograms revealed that IsPDIA6-incubated cells formed a separate cluster from the cells without IsPDIA6 after *B. burgdorferi* stimulation (Fig. 4D and E). We found that 270 genes were differentially expressed (*P* < 0.05 and fold change > 2), 207 genes were upregulated, and 63 genes were downregulated when incubated with IsPDIA6 (Table S2). Many of the upregulated genes encoded cytokines, including IL-17f, Cxcl10, Tnfsf10, IL-27, IL-6, and IFN-γ, and interferon-induced proteins, including Mx1, Mx2, Ifit1, and Ifit2. In addition, C1qa and C1qc gene expression was significantly downregulated by IsPDIA6 (Fig. 4F; Table S2). These data further confirmed the immunomodulation roles of IsPDIA6.

### *IsPDIA6* enzyme active sites analysis

**Fig 5:**
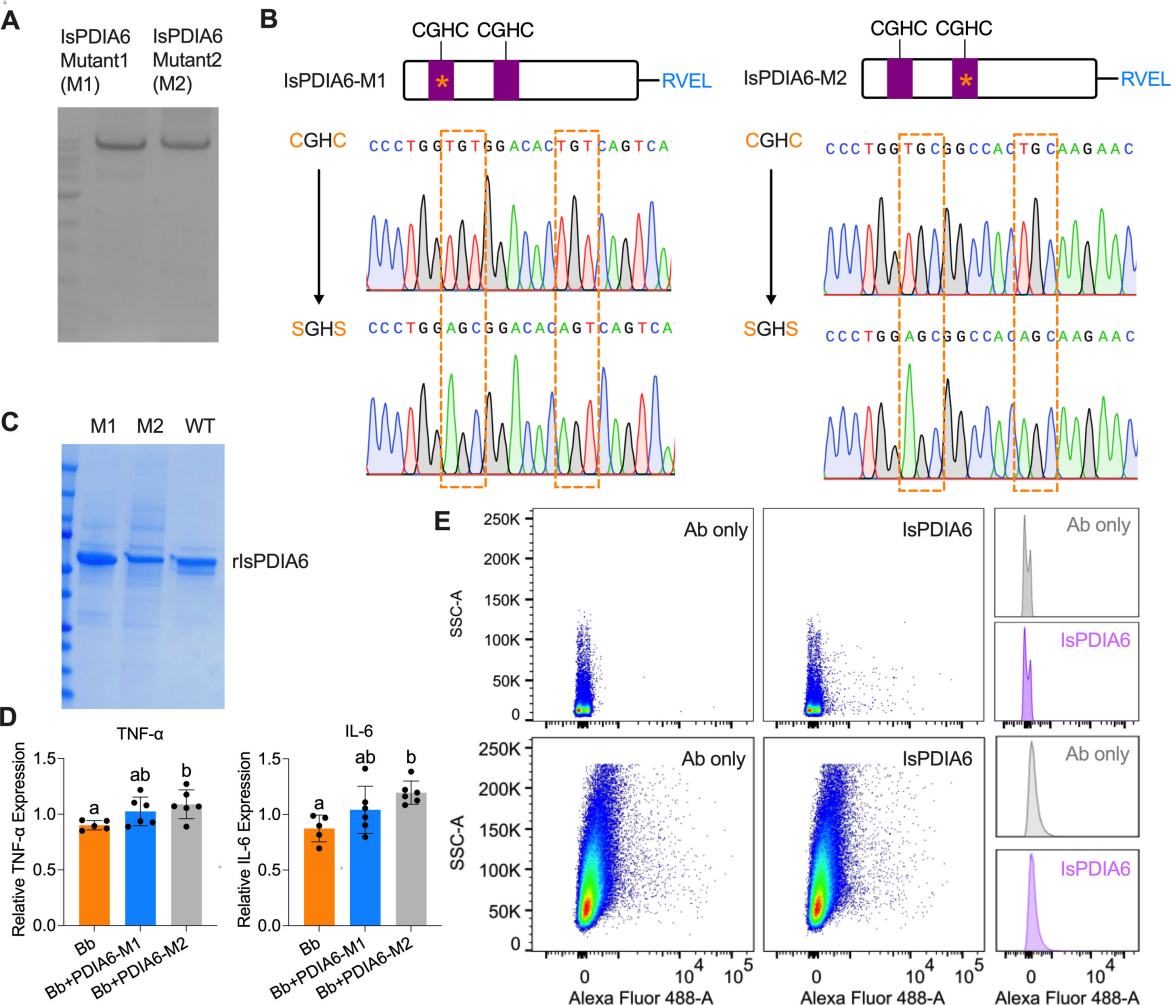
IsPDIA6 enzyme active sites analysis. (**A**) Gel of the clone from site-directed mutagenesis of IsPDIA6, IsPDIA6 mutant 1 (IsPDIA6-M1) and IsPDIA6-M2. (**B**) Sequencing results of two enzyme active site-directed mutagenesis of IsPDIA6 (IsPDIA6-M1 and IsPDIA6-M2). (**C**) The Coomassie Blue SDS-PAGE gel of recombinant IsPDIA6 wild type (WT), IsPDIA6-M1, and IsPDIA6-M2. (**D**) IsPDIA6-M2 significantly induces TNF-α and IL-6 gene expression in splenocytes upon *B. burgdorferi* stimulation; however, IsPDIA6-M1 has no effect on cytokine gene expression compared to the control. (**E**) IsPDIA6 does not bind to the majority of splenocytes (upper panel), and macrophages (lower panel) as analyzed by flow cytometry. The control is splenocytes incubated with Alexa Fluor 488-His antibody. For all the data, each dot represents one biological replicate. Statistical significance was assessed using a non-parametric Mann-Whitney test (*P < 0.05; **P < 0.01; ns, *P* > 0.05) or one-way ANOVA with Tukey’s post hoc test.

As IsPDIA6 has two enzyme active site motifs (CGHC), we further investigated which active site(s) contributes to the immunomodulatory role of IsPDIA6. Two IsPDIA6 mutant clones (IsPDIA6-M1 and IsPDIA6-M2) were made by mutating the individual CGHC motifs to SGHS (Fig. 5A and B). The recombinant protein of the two mutants was further purified (Fig. 5C). The splenocytes were stimulated by *B. burgdorferi,* with or without IsPDIA6-M1 and IsPDIA6-M2 for 6 h. The gene expression of TNF-α and IL-6 was further evaluated. We found that IsPDIA6-M2 still significantly induces TNF-α and IL-6 gene expression in splenocytes upon *B. burgdorferi* stimulation; however, IsPDIA6-M1 has no effect on cytokine gene expression compared to the control (Fig. 5D). This indicates that the first CGHC enzyme active site contributes to the immunomodulatory role of IsPDIA6. As tick salivary proteins may bind to the receptor(s) of host immune cells to regulate gene expression of cytokines and chemokines (23–25), we further examine whether IsPDIA6 could bind to the isolated splenocytes and macrophages by flow-cytometry-based binding assays. rIsPDIA6 cannot readily bind to immune cells (Fig. 5E), suggesting an alternative mechanism, such as cell fusion or interaction with secreted proteins from immune cells.

## DISCUSSION

PDI proteins can catalyze the formation and breakage of disulfide bonds between cysteine residues within proteins as they fold, ensuring that proteins are in a properly folded state in the ER. PDIs can also serve as molecular chaperones for protein synthesis and maturation. Outside the ER, the recruitment of extracellular PDIs has been demonstrated to be essential for cell invasion by diverse intracellular pathogens (5, 6). In contrast to the studies of vertebrate PDIs, an understanding of PDIs in arthropods is limited (26–28). Furthermore, whether vector PDIs are associated with pathogen infectivity has not been examined.

In this study, we investigated the *I. scapularis* PDI gene family and explored their potential influence on *B. burgdorferi*, the most common tick-borne pathogen in North America. Of the newly identified PDIs, *IsPDIA6* gene expression was significantly downregulated in both the tick salivary gland and midgut upon *B. burgdorferi* infection, suggesting that *IsPDIA6* may have a role during *B. burgdorferi* colonization of the tick midgut and/or transmission from tick salivary glands. Through RNAi knockdown of *IsPDIA6*, we found that IsPDIA6 impairs *B. burgdorferi* colonization and enhances *B. burgdorferi* transmission. We further demonstrated that IsPDIA6 can mediate trypsin inhibitor gene expression, potentially contributing to the prevention of *B. burgdorferi* membrane degradation from proteases digestion in tick midgut (19). Indeed, trypsin and trypsin inhibitors influence the colonization and replication of pathogens in vector midgut. For instance, mosquito midgut trypsin activity affects dengue virus titer and replication (29, 30).

IsPDIA6 is also secreted into saliva, and IsPDIA6 antibodies are elicited following a natural tick bite. Therefore, IsPDIA6 is likely to function at the vector-host interface to influence *B. burgdorferi* transmission by the tick. Indeed, tick saliva facilitates blood feeding and enhances pathogen survival and transmission. As one example, a tick C1q protein alters infectivity of *B. burgdorferi* by modulating host IFN-γ production in immune cells (23). In this study, we found the expression of several cytokines/chemokines, including TNF-α, IFN-γ, and IL-6, was induced in the presence of rIsPDIA6. Cytokine/chemokine profiles at the bite sites of mice fed upon by *IsPDIA6* knockdown ticks also showed that IL-6 was significantly downregulated, suggesting that IsPDIA6 mediates inflammatory microenvironment at the bite site and thereby influences *B. burgdorferi* transmission to hosts (25). Typically, PDIs include two catalytically catalytic domains (a and a′) with a CXXC active motif. Based on the site-directed mutagenesis of IsPDIA6, we found the first CGHC enzyme active site contributes to the immunomodulation roles of IsPDIA6. Interestingly, our previous study indicated that, in contrast to IsPDIA6, another *I. scapularis* PDI homolog (PDIA3) enhanced *B. burgdorferi* colonization of the tick but did not influence transmission to the murine host (12). Likewise, while IsPDIA6 seems to have a proinflammatory effect, PDIA3 downregulates several cytokines in the host, including IL-18, IL-1β, IL-4, and IL-17. These results highlight the differential effect/role of PDIs in *I. scapularis*.

In summary, this study represents a detailed investigation of tick PDIs and provides a novel pathway to influence the ability of *B. burgdorferi* to efficiently complete its life cycle. These data suggest that tick PDIs play a role in pathogen colonization and transmission, and this paradigm may be applicable to other vectors of important infectious diseases.

## MATERIALS AND METHODS

### Animals

Six-week-old female C3H/HeN mice were purchased from the Charles River Laboratories. The mice were maintained in a specific pathogen-free (SPF) facility at Yale University. To obtain *B. burgdorferi*-infected mice, the mice were injected subcutaneously with 100 µL of 1 × 10^5^ cells/mL *B. burgdorferi* (strain N40).

### Ticks

SPF *I. scapularis* larvae were obtained from the Oklahoma State University (Stillwater, OK). The larval ticks were fed on pathogen-free or spirochetes-infected mice and allowed to molt to nymphs. All the ticks were maintained at 85% relative humidity with a 14-h light and 10-h dark period at 23°C.

### Spirochetes

The spirochetes *B. burgdorferi* (strain N40) were grown in Barbour-Stoenner-Kelly H complete medium (Sigma-Aldrich, #B8291) in a 33°C setting incubator. The live cell density was determined by dark field microscopy and hemocytometer (INCYTO, #DHC-N01).

### Cells

Drosophila S2 cells (ATCC, #CRL-1963) were grown in Schneider’s Drosophila media (Gibco, #21720-024) with 10% FBS at 28°C. Murine splenocytes were isolated from mice as described in Tang et al. (23). Briefly, the spleens were minced in RPMI 1640 medium (Sigma-Aldrich, #R4130) and forced through a 70-µm cell strainer nylon mesh (Fisherbrand, #22-363-548). After a single wash with PBS and red blood cells lysis with NH_4_Cl, the cells were resuspended in RPMI 1640 medium. RAW264.7 cells (ATCC, #TIB-71) were cultured in DMEM (ThermoFisher, #11965-118) at 37°C.

### Bioinformatic analysis

The homologs of human PDIAs were Blasted in NCBI and VectorBase (https://vectorbase.org/vectorbase/) with six human PDIAs. Signal peptides and their cleavage sites in PDIA proteins were predicted by SignalP 5.0 (https://services.healthtech.dtu.dk/services/SignalP-5.0/). Conserved domains were predicted by the NCBI CD search program (https://www.ncbi.nlm.nih.gov/Structure/cdd/wrpsb.cgi) and InterPro (https://www.ebi.ac.uk/interpro/). Multiple alignment of protein sequences and phylogenetic tree was performed using the Clustal Omega (https://www.ebi.ac.uk/Tools/msa/clustalo/).

### Gene expression evaluation by quantitative real-time PCR

SPF and *B. burgdorferi*-infected *I. scapularis* nymphs were dissected for midguts and salivary glands. The RNA from the dissected tissues was purified by Trizol (Invitrogen, #15596-018) following the manufacturer’s protocol, and cDNA was synthesized using the iScript cDNA Synthesis Kits (Bio-Rad, #1708891). qPCR was performed using iTaq SYBR Green Supermix (Bio-Rad, #1725124) with an initial denaturing step of 2 min at 95°C and 45 cycles. Each cycle consists of 95°C for 20 s, followed by 15 s at 60°C, and 30 s at 72°C. The primer sequences of target genes are shown in Table S3.

### Gene silencing and effects on *B. burgdorferi* acquisition and transmission

To silence the targeted genes, dsRNA primers with T7 promoter sequences were designed (Table S3), and dsRNAs were synthesized using the MEGAscript RNAi Kit (Invitrogen, #AM1626M). To examine the effects of silencing targeted genes on the acquisition of *B. burgdorferi* in tick gut, dsRNA-microinjected pathogen-free *I. scapularis* nymphs were placed on *B. burgdorferi*-infected mice (C3H/HeN) and allowed to feed to repletion. DsRNA of the Aequorea victoria green fluorescent protein was used as a control. The fed ticks were collected for gut dissection. The *B. burgdorferi* burden in the tick gut was quantified by qPCR as described above in “Gene expression evaluation by quantitative real-time PCR.” The *B. burgdorferi* FlaB gene was quantified by extrapolation from a standard curve derived from a series of known DNA dilutions of flaB gene, and data were normalized to tick actin. The knockdown efficiency of targeted genes was estimated with the 2^−∆∆CT^ method (31) using the reference gene actin.

To examine the effects of silencing targeted genes on the transmission of *B. burgdorferi*, *B. burgdorferi*-infected nymphs were injected into the tick body with dsRNA as described in the previous paragraph. A group of four *GFP* or *IsPDIA6* dsRNA-injected *B. burgdorferi*-infected nymphs were placed on each C3H/HeN mouse and allowed to feed to repletion. After 7, 14, and 21 days of *B. burgdorferi* infection, the mice were anesthetized, and skin was aseptically punch biopsied, and the spirochete burden was assessed by qPCR as described above in “Gene expression evaluation by quantitative real-time PCR.”

### Purification of recombinant proteins

The IsPDIA6 gene full length without signal peptide was PCR amplified from tick nymph cDNA using the primer pair listed in Table S3. For protein expression and purification using the *Drosophila* expression system, the fragment was cloned into the *Bgl*II and *Xho*I sites of the pMT/BiP/V5-His vector (Invitrogen, #V413020). The recombinant protein was expressed and purified from the supernatant by TALON metal affinity resin (Clontech, #635606) and eluted with 150 mM imidazole. The eluted samples were filtered through a 0.22 µm filter and concentrated with a 30 kDa concentrator (MilliporeSigma, # UFC9030) by centrifugation at 4°C. Recombinant protein purities were assessed by SDS-PAGE using 4%–20% Mini-Protean TGX gels (Bio-Rad, #4561094) and quantified using the BCA Protein Estimation Kit (ThermoFisher Scientific, #23225).

### Mouse immunization and antiserum preparation

To obtain murine anti-IsPDIA6 serum, C3H/HeN mice were first immunized with 10 µg of rIsPDIA6 emulsified in complete Freund’s adjuvant (Thermo Fisher Scientific, #77140). Then the mice were immunized with two boosts of the antigen and incomplete Freund’s adjuvant (Thermo Fisher Scientific, #77145) every 2 weeks. Blood was collected from the immunized mice, and the serum was obtained by centrifugation. The antibody titers in serum were further tested by ELISA and western blot to confirm antigen-specific antibodies.

### Western blot to detect IsPDIA6 protein in tick salivary gland and midgut

To detect IsPDIA6 protein in the tick salivary gland and midgut, the dissected tick tissues were lysed with RIPA Lysis and Extraction Buffer (Thermo Scientific, # 89900). The samples were first separated by SDS-PAGE and then transferred onto a 0.45-µm-pore-size polyvinylidene difluoride membrane (Bio-Rad, #1620177) and processed for immunoblotting. The blots were blocked in 1% non-fat milk in PBS for 60 min. Mouse polyclonal anti-IsPDIA6 antiserum was diluted in 0.05% PBST at 1:500 and incubated with the blots for 1 h at room temperature or 4°C overnight. HRP-conjugated secondary antibody (1:2,500, Invitrogen, #62-6520) was diluted in PBST and incubated for 1 h at room temperature. After washing with PBST, the immunoblots were imaged and quantified with an LI-COR Odyssey imaging system. All the SDS-PAGE gels were run under reducing conditions.

### Western blot to detect IsPDIA6 antibody in mammalian serum

To examine if IsPDIA6 antibodies are elicited by natural tick bites in mammalian serum, western blots of rIsPDIA6 were probed with naive serum and serum from guinea pig bitten by ticks. The SDS-PAGE gels were run under reducing conditions. The western blot was conducted as described in the previous paragraph.

### Flow cytometry to examine *B. burgdorferi*-IsPDIA6 interaction

*B. burgdorferi* was cultured to a density of 10^6^–10^7^ cells/mL and harvested at 5,000 × *g* for 15 min. Cells were washed twice with PBS and then blocked in 1% BSA for 1 h at 4°C. After spin, the pellet was suspended and incubated with rIsPDIA6 at 4°C for 2 h. After a co-incubation, spirochetes were washed three times with PBS and fixed in 2% PFA. After washing, the spirochetes were probed anti-6×-His monoclonal antibody conjugated to Alexa Fluor 488 (Invitrogen, #MA1-21315-488) and run through BD LSRII (BD bioscience). The data were then analyzed by FlowJo.

### Cell viability assay

To test if IsPDIA6 has effect on *B. burgdorferi* viability, 10^6^ cells/mL *B. burgdorferi* were incubated with 1× PBS, 10, 20, and 40 mg/mL IsPDIA6 at 33°C for 48 h in a final volume of 250 µL. The viability of *B. burgdorferi* was determined as described in Tang et al. (23) using BacTiter-Glo Microbial Cell Viability Assay Kit (Promega, #G8230), which is based on quantitation of the ATP present by measuring luminescence.

### RNA-seq analysis

To understand the global gene expression of ticks after silencing *IsPDIA6*, ds *GFP-* and ds *IsPDIA6*-microinjected SPF *I. scapularis* nymphs were placed on *B. burgdorferi*-infected C3H/HeN mice and allowed to feed to repletion. Then, the ticks were collected for gut dissection, and the total RNA was purified as described above in “Gene expression evaluation by quantitative real-time PCR.” RNA was submitted for library preparation using TruSeq (Illumina, San Diego, CA, USA) and sequenced using Illumina HiSeq 2500 by paired-end sequencing at the Yale Center for Genome Analysis. All the RNA-seq analyses were performed using Partek Genomics Flow software (St. Louis, MO, USA). RNA-seq raw data were trimmed and aligned to the tick genome using HISAT2 (32). The aligned reads were then quantified and normalized by dividing the gene counts by the total number of reads followed by the addition of a small offset (0.0001). The counts generated from three biological replicates of each treatment were processed by DESeq2 (33). Principal components analysis and gene expression heatmap were performed using default parameters for the determination of the component number, with all components contributing equally in Partek Flow. Volcano plot was performed on the genes that were differentially expressed across the conditions (*P* < 0.05, FDR < 0.05, and fold change ≥ 2 for each comparison).

### Effects of IsPDIA6 on host cytokine gene expression

We determined the effects of rIsPDIA6 on host cytokine gene expression. The murine splenocytes were isolated as described in our previous study (23). The isolated cells or RAW264.7 cells were stimulated by 10^6^ *B. burgdorferi* with or without 1 mg/mL rIsPDIA6 for 6 h. The cells and supernatant were harvested, and total RNA extraction and gene expression by qPCR and RNA-seq were performed as described above in “RNA-Seq analysis.”

### Site-directed mutagenesis

To obtain two IsPDIA6 mutant clones, the primers were designed according to the targeted bases (Table S3). The IsPDIA6 mutant clones were PCR amplified from pEZT-IsPDIA6 or pMT-IsPDIA6 plasmids using Q5 Hot Start High-Fidelity 2× Master Mix from Q5 Site-Directed Mutagenesis Kit (New England Biolabs, #E0554S). The IsPDIA6 mutant plasmids were obtained after transformation. The recombinant protein was then expressed and purified as described above in “Purification of recombinant proteins.”

### Flow cytometry to examine immune cell-IsPDIA6 interaction

RAW264.7 cells or splenocytes (10^6^ cells/mL) were washed twice with PBS and then blocked in 1% BSA for 1 h at 4°C. After spin, the pellet was suspended and incubated with rIsPDIA6 at 4°C for 2 h. After a co-incubation, cells were washed three times with PBS and fixed in 2% PFA. After washing, the cells were probed anti-6×-His monoclonal antibody conjugated to Alexa Fluor 488 (Invitrogen, #MA1-21315-488) and run through BD LSRII (BD bioscience). The data were then analyzed by FlowJo.

### Statistical analysis

Statistical significance of differences observed in experimental and control groups was analyzed using GraphPad Prism version 8.0 (GraphPad Software, Inc., San Diego, CA) or IBM SPSS Statistics version 29.0.2.0 (Armonk, NY: IBM Corp). Non-parametric Mann-Whitney test, unpaired *t* test, or one-way ANOVA with Tukey’s post hoc test was utilized to compare the mean values of control and tested groups, and *P* < 0.05 was considered significant.

## Data Availability

All data are available in the main text or the supplemental material. All the RNA-seq data sets used in this study are available in the supplemental material or the National Center for Biotechnology Information (NCBI) Sequence Read Archive (GSE273486 and GSE273568).

## SUPPLEMENTAL MATERIAL

Supplemental material mbio.01754-24-s0001.docx Supplemental figures and tables.

ASM does not own the copyrights to Supplemental Material that may be linked to, or accessed through, an article. The authors have granted ASM a non-exclusive, world-wide license to publish the Supplemental Material files. Please contact the corresponding author directly for reuse.
