# Supplementary material for: Dual roles for a tick protein disulfide isomerase during the life cycle of the Lyme disease agent
Source: mBio. 2024 Oct 29;15(12):e01754-24. doi: 10.1128/mbio.01754-24 (PMC11633212; doi:10.1128/mbio.01754-24)
Supplement: Supplemental material — Supplemental figures and tables. [file mbio.01754-24-s0001.docx]

**Figure S1. Multiple sequence alignment of five *Ixodes scapularis* PDIAs**. (*) indicates positions that have a single, fully conserved residue. (:) indicates conservation between groups of strongly similar properties. (.) indicates conservation between groups of weakly similar properties.


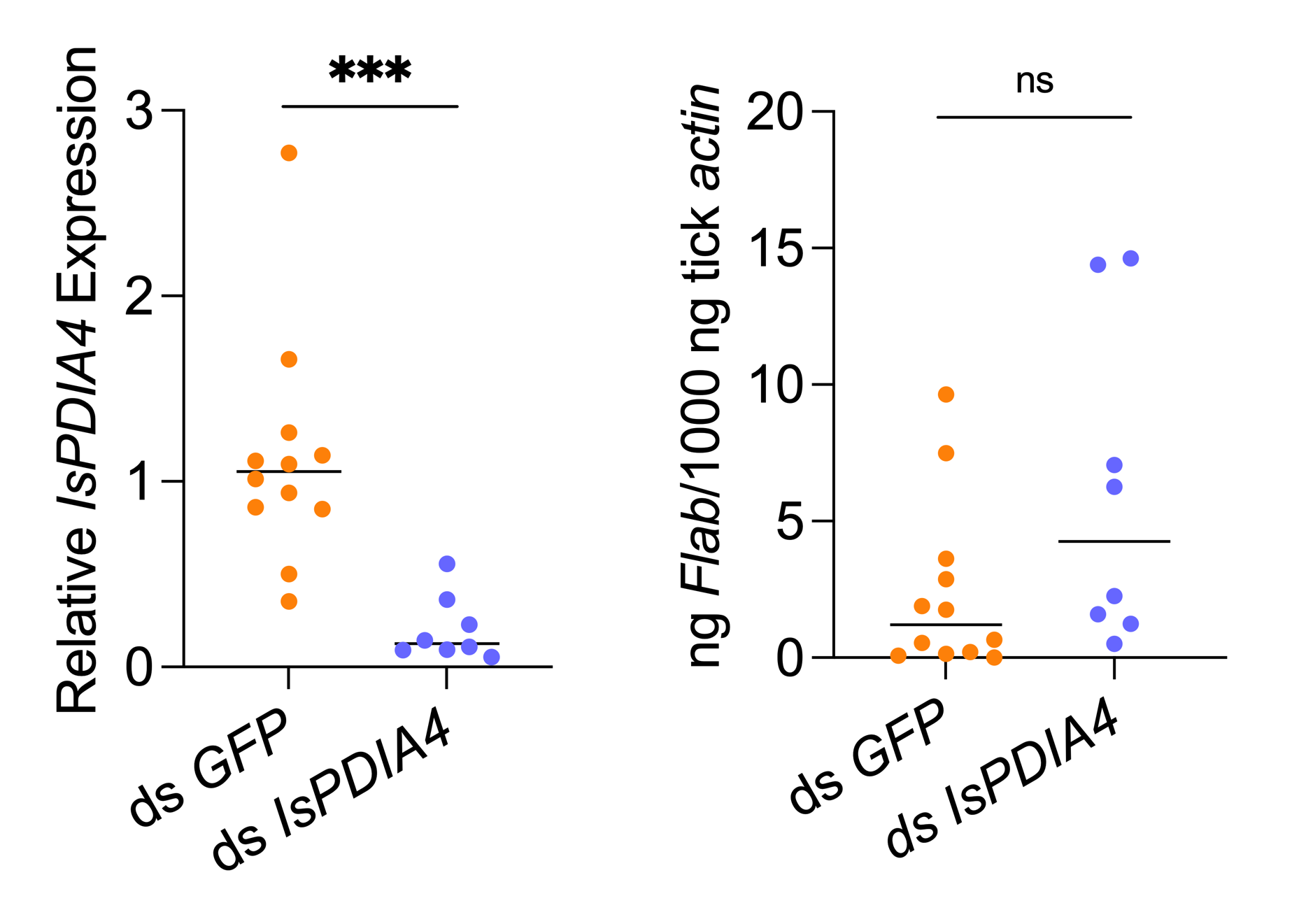


**Figure S2.** Silencing of *IsPDIA4* has no effect on the *B. burgdorferi* burden in nymphal tick guts. Each dot represents one biological replicate. Statistical significance was assessed using a non-parametric Mann-Whitney test (****p* < 0.001; ns, *p* > 0.05).


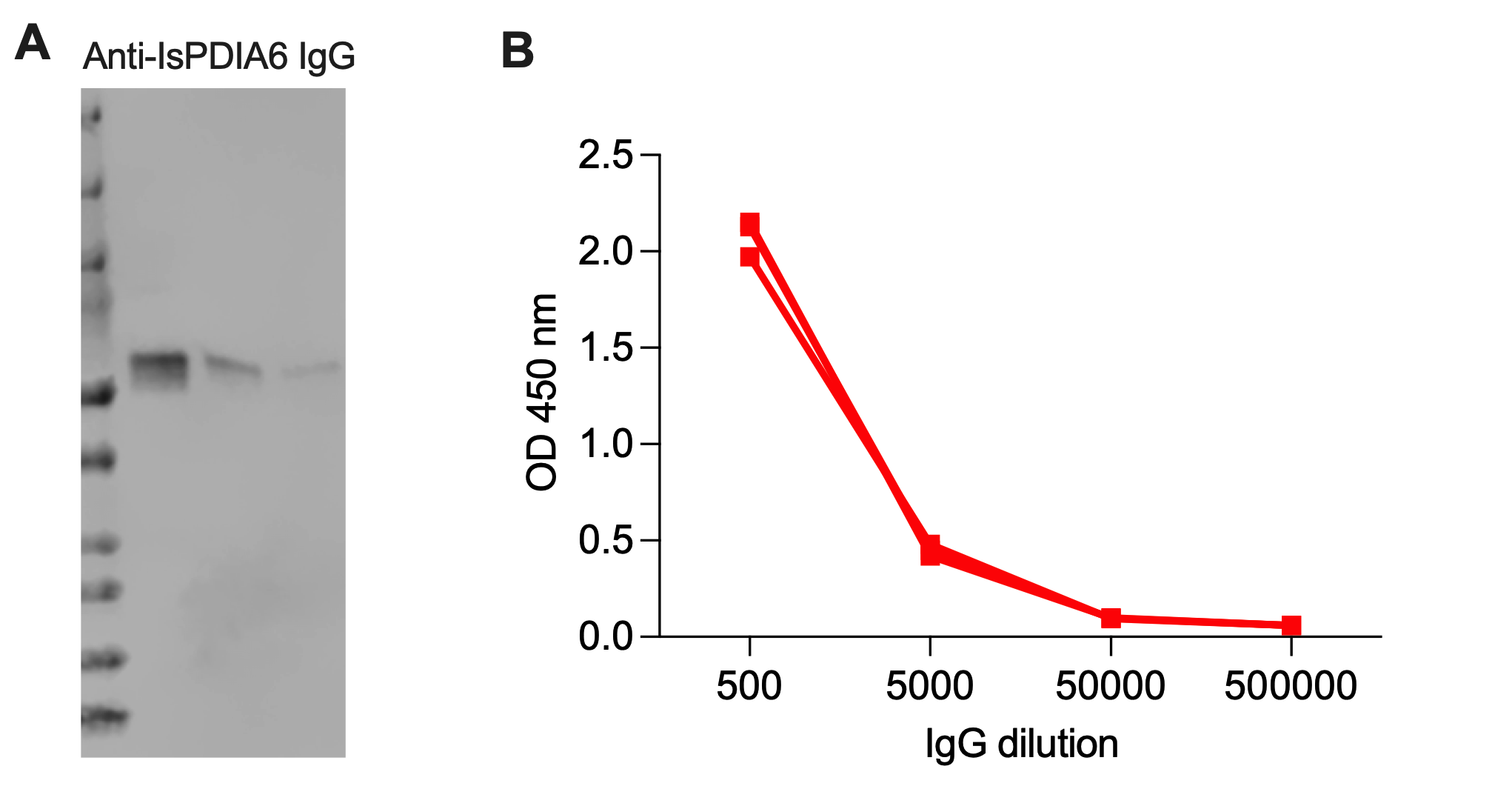


**Figure S3. Antibody titer of IsPDIA6.** (A) IgG of sera from IsPDIA6-immunized mice recognized rIsPDIA6 as revealed by western blot. (B) IgG of sera from IsPDIA6-immunized mice recognized rIsPDIA6 as revealed by ELISA.


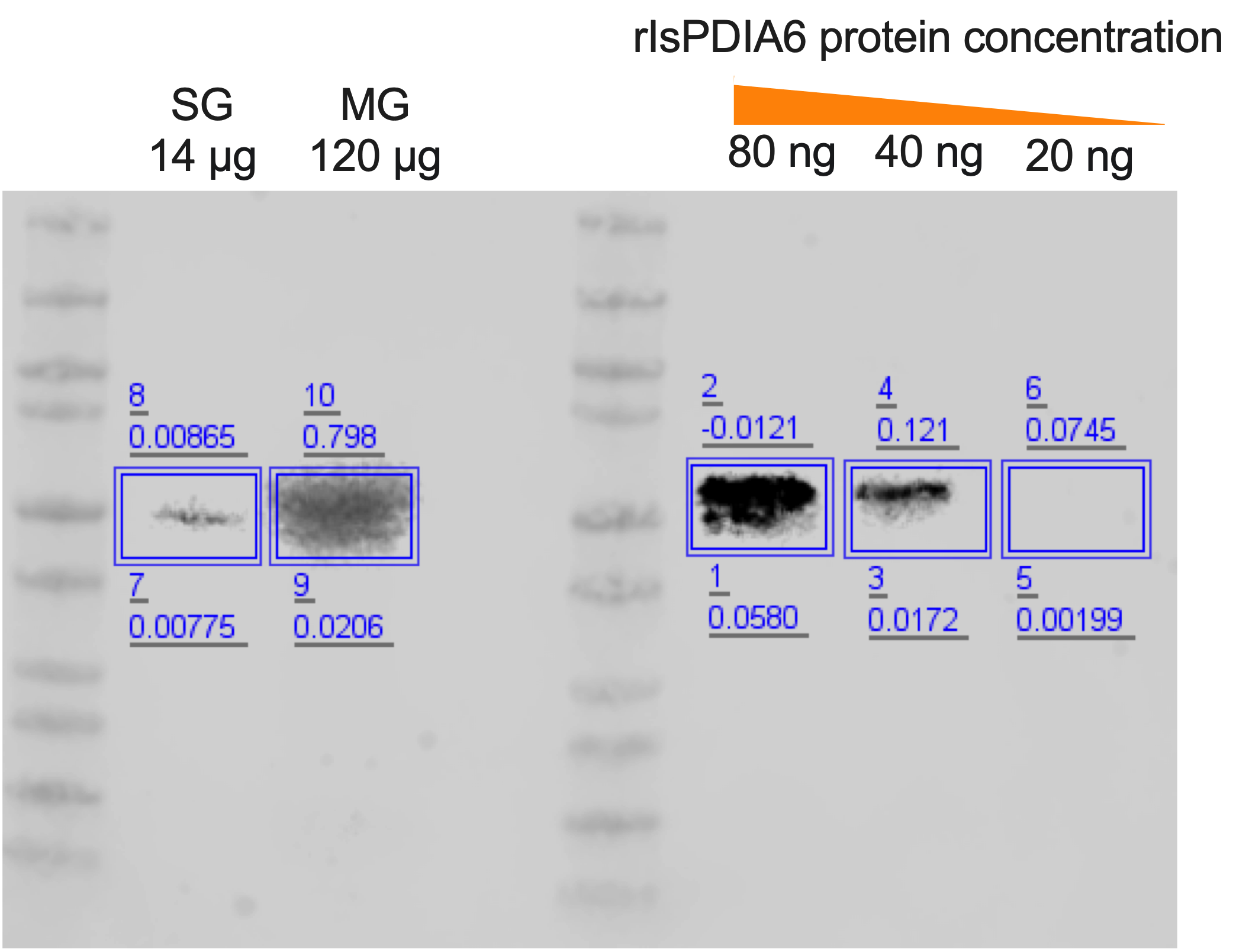


**Figure S4. Quantification of IsPDIA6 in fed tick salivary gland (SG) and midgut (MG).**


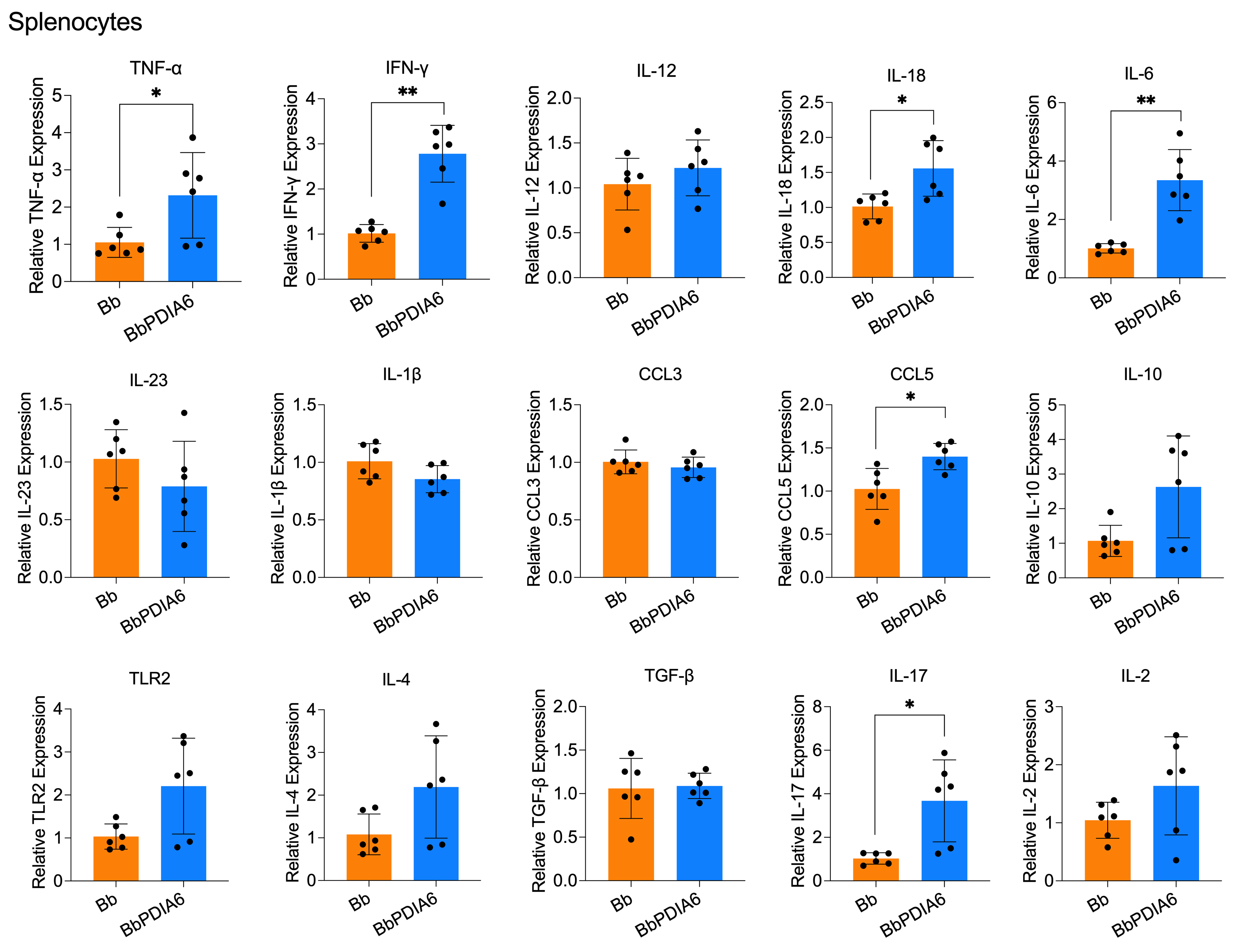


**Figure S5. The effects of IsPDIA6 on gene expression of cytokines and chemokines**

**in splenocytes upon *B. burgdorferi* stimulation.**


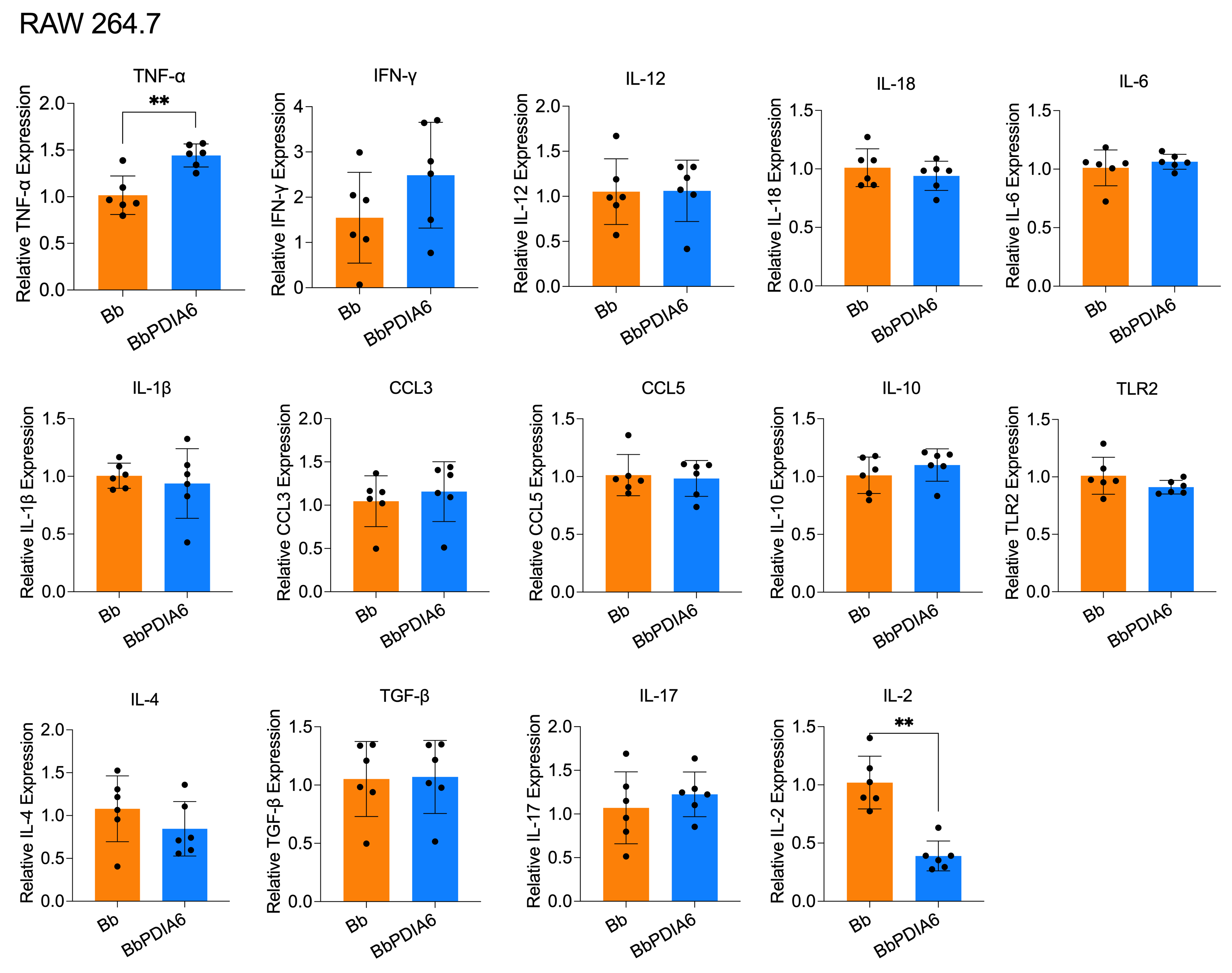


**Figure S6. The effects of IsPDIA6 on gene expression of cytokines and chemokines**

**in RAW264.7 macrophages upon *B. burgdorferi* stimulation.**

**Table S1. Differentially expressed genes of comparison between ds *GFP* and ds *IsPDIA6* injected-ticks.**

| **Feature ID** | **Description** | **P-value** | **Fold change** | **LSMean(ds PDIA6)** | **LSMean(ds GFP)** |
| --- | --- | --- | --- | --- | --- |
| ISCW012412 | Kunitz domain protein, putative | 0.0000 | 13.3066 | 264.3187 | 19.8637 |
| ISCW015846 | Neurotrimin, putative | 0.0000 | 9.4952 | 22.4333 | 2.3626 |
| ISCW004112 | uncharacterized protein | 0.0000 | 5.2280 | 45.3728 | 8.6788 |
| ISCW000669 | uncharacterized protein | 0.0000 | 4.5564 | 12.0199 | 2.6380 |
| ISCW004395 | uncharacterized protein | 0.0000 | 2.4351 | 49.3458 | 20.2641 |
| ISCW012333 | uncharacterized protein | 0.0000 | 2.3229 | 170.9855 | 73.6076 |
| ISCW007836 | Trypsin inhibitor, putative | 0.0000 | 2.1917 | 216.6578 | 98.8530 |
| ISCW004397 | tetraspanin, putative | 0.0000 | 1.9871 | 547.1408 | 275.3403 |
| ISCW014857 | ATP binding protein, putative | 0.0000 | -2.9936 | 33.3918 | 99.9616 |
| ISCW002080 | Protein disulfide isomerase 1, putative | 0.0000 | -5.2425 | 34.0085 | 178.2883 |

**Table S2. Differentially expressed genes of transcriptome data from splenocytes that were stimulated by *B. burgdorferi*, with or without IsPDIA6.**

| **Gene ID** | **Gene name** | **P-value** | **Fold change** |
| --- | --- | --- | --- |
| ENSMUSG00000000385 | Tmprss2 | 0.0000 | 10.1842 |
| ENSMUSG00000082576 | Gm12058 | 0.0070 | 8.8016 |
| ENSMUSG00000041872 | Il17f | 0.0346 | 7.6106 |
| ENSMUSG00000078173 | Lenep | 0.0076 | 7.4553 |
| ENSMUSG00000105093 | Gm17815 | 0.0179 | 7.1983 |
| ENSMUSG00000103000 | Gm37900 | 0.0093 | 6.7258 |
| ENSMUSG00000114644 | Gm47243 | 0.0010 | 6.5902 |
| ENSMUSG00000042942 | Greb1l | 0.0160 | 5.7789 |
| ENSMUSG00000078616 | Trim30c | 0.0000 | 5.3421 |
| ENSMUSG00000082778 | Gm15191 | 0.0294 | 5.1796 |
| ENSMUSG00000000386 | Mx1 | 0.0000 | 4.9727 |
| ENSMUSG00000086327 | Slfn5os | 0.0000 | 4.9616 |
| ENSMUSG00000036687 | Tmem184a | 0.0336 | 4.9107 |
| ENSMUSG00000023341 | Mx2 | 0.0000 | 4.8838 |
| ENSMUSG00000095866 | Ighv2-4 | 0.0355 | 4.8496 |
| ENSMUSG00000110326 | Gm45378 | 0.0357 | 4.7157 |
| ENSMUSG00000089945 | Pakap | 0.0365 | 4.6948 |
| ENSMUSG00000054404 | Slfn5 | 0.0000 | 4.4994 |
| ENSMUSG00000108500 | Gm45033 | 0.0213 | 4.4966 |
| ENSMUSG00000105979 | Gm43411 | 0.0042 | 4.3979 |
| ENSMUSG00000039236 | Isg20 | 0.0000 | 4.3677 |
| ENSMUSG00000097569 | Gm26640 | 0.0071 | 4.1293 |
| ENSMUSG00000118671 | Eppk1 | 0.0127 | 4.1290 |
| ENSMUSG00000044703 | Phf11a | 0.0000 | 4.1286 |
| ENSMUSG00000027931 | Npr1 | 0.0213 | 4.1030 |
| ENSMUSG00000108353 | Gm45205 | 0.0214 | 4.0575 |
| ENSMUSG00000041827 | Oasl1 | 0.0000 | 4.0418 |
| ENSMUSG00000087477 | Gm13822 | 0.0139 | 3.9998 |
| ENSMUSG00000034842 | Art3 | 0.0003 | 3.9576 |
| ENSMUSG00000120265 | Gm26698 | 0.0183 | 3.9201 |
| ENSMUSG00000111160 | Gm48855 | 0.0348 | 3.8891 |
| ENSMUSG00000062488 | Ifit3b | 0.0000 | 3.8658 |
| ENSMUSG00000095366 | Gm21860 | 0.0177 | 3.8598 |
| ENSMUSG00000032690 | Oas2 | 0.0000 | 3.8429 |
| ENSMUSG00000074896 | Ifit3 | 0.0000 | 3.8255 |
| ENSMUSG00000078954 | Arhgap8 | 0.0006 | 3.8163 |
| ENSMUSG00000111913 | Gm49751 | 0.0000 | 3.8079 |
| ENSMUSG00000107197 | Gm43312 | 0.0117 | 3.7906 |
| ENSMUSG00000020641 | Rsad2 | 0.0000 | 3.7720 |
| ENSMUSG00000106940 | Gm42930 | 0.0226 | 3.7583 |
| ENSMUSG00000050395 | Tnfsf15 | 0.0000 | 3.7336 |
| ENSMUSG00000074577 | Ripor3 | 0.0274 | 3.7154 |
| ENSMUSG00000028037 | Ifi44 | 0.0000 | 3.6723 |
| ENSMUSG00000109729 | Gm45418 | 0.0485 | 3.5803 |
| ENSMUSG00000006930 | Hap1 | 0.0001 | 3.5578 |
| ENSMUSG00000105466 | Gm42998 | 0.0362 | 3.5279 |
| ENSMUSG00000085802 | Gm16059 | 0.0129 | 3.5142 |
| ENSMUSG00000031765 | Mt1 | 0.0001 | 3.5138 |
| ENSMUSG00000031639 | Tlr3 | 0.0000 | 3.5101 |
| ENSMUSG00000056148 | Rdh9 | 0.0492 | 3.5053 |
| ENSMUSG00000029392 | Rilpl1 | 0.0000 | 3.4887 |
| ENSMUSG00000090881 | Phf11 | 0.0002 | 3.4757 |
| ENSMUSG00000120804 | Gm56993 | 0.0321 | 3.4483 |
| ENSMUSG00000032661 | Oas3 | 0.0000 | 3.3823 |
| ENSMUSG00000078143 | Gm17344 | 0.0238 | 3.3739 |
| ENSMUSG00000107075 | Gm43068 | 0.0000 | 3.3732 |
| ENSMUSG00000030107 | Usp18 | 0.0000 | 3.3474 |
| ENSMUSG00000034855 | Cxcl10 | 0.0000 | 3.3383 |
| ENSMUSG00000070803 | Cited4 | 0.0357 | 3.3103 |
| ENSMUSG00000035692 | Isg15 | 0.0000 | 3.3039 |
| ENSMUSG00000102222 | Pcdhga10 | 0.0264 | 3.2888 |
| ENSMUSG00000045932 | Ifit2 | 0.0000 | 3.2862 |
| ENSMUSG00000086213 | A330040F15Rik | 0.0000 | 3.2781 |
| ENSMUSG00000121201 | Gm57239 | 0.0338 | 3.2583 |
| ENSMUSG00000034459 | Ifit1 | 0.0000 | 3.2560 |
| ENSMUSG00000114605 | C130051F05Rik | 0.0393 | 3.2306 |
| ENSMUSG00000020638 | Cmpk2 | 0.0000 | 3.2205 |
| ENSMUSG00000047511 | Or2v2 | 0.0000 | 3.2038 |
| ENSMUSG00000108112 | Gm45193 | 0.0000 | 3.1626 |
| ENSMUSG00000020135 | Apc2 | 0.0093 | 3.1604 |
| ENSMUSG00000108752 | Gm45191 | 0.0110 | 3.1479 |
| ENSMUSG00000031257 | Nox1 | 0.0253 | 3.1359 |
| ENSMUSG00000091649 | Phf11b | 0.0000 | 3.1072 |
| ENSMUSG00000114761 | Gm47242 | 0.0000 | 3.0871 |
| ENSMUSG00000096078 | Ighv1-62-2 | 0.0129 | 3.0819 |
| ENSMUSG00000052776 | Oas1a | 0.0000 | 3.0744 |
| ENSMUSG00000026896 | Ifih1 | 0.0000 | 3.0574 |
| ENSMUSG00000111118 | Gm6545 | 0.0000 | 3.0485 |
| ENSMUSG00000029798 | Herc6 | 0.0000 | 3.0311 |
| ENSMUSG00000103937 | Gm37186 | 0.0299 | 3.0158 |
| ENSMUSG00000044122 | Proca1 | 0.0297 | 2.9838 |
| ENSMUSG00000000204 | Slfn4 | 0.0000 | 2.9661 |
| ENSMUSG00000014773 | Dll1 | 0.0000 | 2.9635 |
| ENSMUSG00000068245 | Phf11d | 0.0000 | 2.9141 |
| ENSMUSG00000039103 | Nexn | 0.0370 | 2.9062 |
| ENSMUSG00000116639 | Gm49730 | 0.0000 | 2.9012 |
| ENSMUSG00000031762 | Mt2 | 0.0203 | 2.8966 |
| ENSMUSG00000037855 | Zfp365 | 0.0000 | 2.8914 |
| ENSMUSG00000025492 | Ifitm3 | 0.0000 | 2.8877 |
| ENSMUSG00000095429 | Ighv5-12 | 0.0332 | 2.8849 |
| ENSMUSG00000037921 | Ddx60 | 0.0000 | 2.8825 |
| ENSMUSG00000048534 | Jaml | 0.0000 | 2.8707 |
| ENSMUSG00000017830 | Dhx58 | 0.0000 | 2.8519 |
| ENSMUSG00000105008 | Gm43652 | 0.0479 | 2.8189 |
| ENSMUSG00000054083 | Capn12 | 0.0265 | 2.8140 |
| ENSMUSG00000025743 | Sdc3 | 0.0000 | 2.8024 |
| ENSMUSG00000102776 | Gm38162 | 0.0031 | 2.8000 |
| ENSMUSG00000102856 | Gm37084 | 0.0037 | 2.7984 |
| ENSMUSG00000109408 | A930037H05Rik | 0.0000 | 2.7947 |
| ENSMUSG00000055170 | Ifng | 0.0000 | 2.7626 |
| ENSMUSG00000103094 | Gm37558 | 0.0370 | 2.7473 |
| ENSMUSG00000091144 | Phf11c | 0.0000 | 2.7253 |
| ENSMUSG00000102153 | Gm37474 | 0.0282 | 2.7175 |
| ENSMUSG00000070327 | Rnf213 | 0.0000 | 2.7169 |
| ENSMUSG00000092349 | Smim40 | 0.0476 | 2.7048 |
| ENSMUSG00000021338 | Carmil1 | 0.0000 | 2.7021 |
| ENSMUSG00000017740 | Slc12a5 | 0.0485 | 2.6980 |
| ENSMUSG00000029925 | Tbxas1 | 0.0022 | 2.6973 |
| ENSMUSG00000046378 | Asphd1 | 0.0351 | 2.6783 |
| ENSMUSG00000103621 | Gm38366 | 0.0002 | 2.6401 |
| ENSMUSG00000078349 | AW011738 | 0.0000 | 2.6285 |
| ENSMUSG00000060519 | Tor3a | 0.0000 | 2.6183 |
| ENSMUSG00000000392 | Fap | 0.0118 | 2.6183 |
| ENSMUSG00000057596 | Trim30d | 0.0000 | 2.6138 |
| ENSMUSG00000069911 | Insyn2b | 0.0171 | 2.5930 |
| ENSMUSG00000029561 | Oasl2 | 0.0000 | 2.5822 |
| ENSMUSG00000090559 | Gm17137 | 0.0125 | 2.5791 |
| ENSMUSG00000091199 | Gm2619 | 0.0158 | 2.5740 |
| ENSMUSG00000097129 | 4930507D05Rik | 0.0226 | 2.5728 |
| ENSMUSG00000106959 | Gm42548 | 0.0147 | 2.5654 |
| ENSMUSG00000103105 | Gm18752 | 0.0000 | 2.5507 |
| ENSMUSG00000090628 | Gm17083 | 0.0355 | 2.5445 |
| ENSMUSG00000031596 | Slc7a2 | 0.0000 | 2.5318 |
| ENSMUSG00000104030 | 5330406M23Rik | 0.0287 | 2.5317 |
| ENSMUSG00000106636 | Gm43813 | 0.0001 | 2.5296 |
| ENSMUSG00000111971 | Gm48678 | 0.0458 | 2.5211 |
| ENSMUSG00000025498 | Irf7 | 0.0000 | 2.5182 |
| ENSMUSG00000044145 | 1810024B03Rik | 0.0128 | 2.5128 |
| ENSMUSG00000034872 | Gipc3 | 0.0000 | 2.5083 |
| ENSMUSG00000024079 | Eif2ak2 | 0.0000 | 2.5024 |
| ENSMUSG00000071856 | Mcc | 0.0313 | 2.5000 |
| ENSMUSG00000018986 | Slfn3 | 0.0000 | 2.4974 |
| ENSMUSG00000000248 | Clec2g | 0.0026 | 2.4965 |
| ENSMUSG00000094796 | BC147527 | 0.0000 | 2.4780 |
| ENSMUSG00000034452 | Slc24a1 | 0.0164 | 2.4767 |
| ENSMUSG00000073491 | Ifi213 | 0.0000 | 2.4710 |
| ENSMUSG00000074899 | Sptbn5 | 0.0280 | 2.4683 |
| ENSMUSG00000063445 | Nmral1 | 0.0000 | 2.4533 |
| ENSMUSG00000114196 | Gm47547 | 0.0258 | 2.4472 |
| ENSMUSG00000104988 | Gm43622 | 0.0001 | 2.4415 |
| ENSMUSG00000045377 | Tmem88 | 0.0277 | 2.4390 |
| ENSMUSG00000108291 | Gm44292 | 0.0229 | 2.4358 |
| ENSMUSG00000058163 | Gm5431 | 0.0000 | 2.4273 |
| ENSMUSG00000083811 | Gm13071 | 0.0443 | 2.4251 |
| ENSMUSG00000030921 | Trim30a | 0.0000 | 2.4193 |
| ENSMUSG00000105550 | Gm35585 | 0.0105 | 2.4072 |
| ENSMUSG00000073489 | Ifi204 | 0.0000 | 2.4049 |
| ENSMUSG00000086866 | 4930512H18Rik | 0.0114 | 2.3799 |
| ENSMUSG00000102960 | Gm37943 | 0.0317 | 2.3704 |
| ENSMUSG00000027570 | Col9a3 | 0.0001 | 2.3615 |
| ENSMUSG00000038507 | Parp12 | 0.0000 | 2.3604 |
| ENSMUSG00000079492 | Gm11127 | 0.0201 | 2.3475 |
| ENSMUSG00000070388 | Fbxo39 | 0.0140 | 2.3412 |
| ENSMUSG00000066677 | Ifi208 | 0.0000 | 2.3383 |
| ENSMUSG00000039304 | Tnfsf10 | 0.0000 | 2.3164 |
| ENSMUSG00000001166 | Oas1c | 0.0000 | 2.3145 |
| ENSMUSG00000110993 | Gm47963 | 0.0329 | 2.3134 |
| ENSMUSG00000104383 | Gm37553 | 0.0179 | 2.3120 |
| ENSMUSG00000040483 | Xaf1 | 0.0000 | 2.3113 |
| ENSMUSG00000108447 | Gm44567 | 0.0063 | 2.3113 |
| ENSMUSG00000071350 | Setdb2 | 0.0000 | 2.3089 |
| ENSMUSG00000033880 | Lgals3bp | 0.0000 | 2.3058 |
| ENSMUSG00000090080 | Gm15872 | 0.0395 | 2.2949 |
| ENSMUSG00000052749 | Trim30b | 0.0000 | 2.2891 |
| ENSMUSG00000040296 | Rigi | 0.0000 | 2.2864 |
| ENSMUSG00000002307 | Daxx | 0.0000 | 2.2833 |
| ENSMUSG00000069793 | Slfn9 | 0.0000 | 2.2795 |
| ENSMUSG00000085604 | Dhx58os | 0.0073 | 2.2745 |
| ENSMUSG00000037849 | Ifi206 | 0.0000 | 2.2580 |
| ENSMUSG00000082154 | Gm16464 | 0.0000 | 2.2576 |
| ENSMUSG00000035208 | Slfn8 | 0.0000 | 2.2414 |
| ENSMUSG00000042726 | Trafd1 | 0.0000 | 2.2381 |
| ENSMUSG00000044701 | Il27 | 0.0040 | 2.2356 |
| ENSMUSG00000086231 | Rapgef4os3 | 0.0376 | 2.2243 |
| ENSMUSG00000107017 | Gm43196 | 0.0001 | 2.2145 |
| ENSMUSG00000028012 | Rrh | 0.0341 | 2.2105 |
| ENSMUSG00000102964 | 9430034N14Rik | 0.0000 | 2.2044 |
| ENSMUSG00000067297 | Ifit1bl2 | 0.0000 | 2.1821 |
| ENSMUSG00000097820 | E530011L22Rik | 0.0475 | 2.1750 |
| ENSMUSG00000111390 | Gm48796 | 0.0485 | 2.1615 |
| ENSMUSG00000070501 | Ifi214 | 0.0000 | 2.1585 |
| ENSMUSG00000112806 | Gm48146 | 0.0359 | 2.1571 |
| ENSMUSG00000001123 | Lgals9 | 0.0000 | 2.1520 |
| ENSMUSG00000035285 | Nat14 | 0.0306 | 2.1327 |
| ENSMUSG00000079339 | Ifit1bl1 | 0.0000 | 2.1293 |
| ENSMUSG00000039997 | Ifi203 | 0.0000 | 2.1176 |
| ENSMUSG00000041936 | Agrn | 0.0000 | 2.1144 |
| ENSMUSG00000025746 | Il6 | 0.0000 | 2.1114 |
| ENSMUSG00000053835 | H2-T24 | 0.0000 | 2.1030 |
| ENSMUSG00000046718 | Bst2 | 0.0000 | 2.1019 |
| ENSMUSG00000027514 | Zbp1 | 0.0000 | 2.0894 |
| ENSMUSG00000062007 | Hsh2d | 0.0000 | 2.0864 |
| ENSMUSG00000009035 | Tmem184b | 0.0000 | 2.0862 |
| ENSMUSG00000027580 | Helz2 | 0.0000 | 2.0854 |
| ENSMUSG00000037593 | Rskr | 0.0327 | 2.0766 |
| ENSMUSG00000043263 | Ifi209 | 0.0000 | 2.0745 |
| ENSMUSG00000112895 | Gm47567 | 0.0114 | 2.0713 |
| ENSMUSG00000036381 | P2ry14 | 0.0019 | 2.0658 |
| ENSMUSG00000033355 | Rtp4 | 0.0000 | 2.0644 |
| ENSMUSG00000106734 | Gm20559 | 0.0000 | 2.0604 |
| ENSMUSG00000121434 | --- | 0.0449 | 2.0591 |
| ENSMUSG00000026536 | Ifi211 | 0.0000 | 2.0488 |
| ENSMUSG00000032344 | Cgas | 0.0000 | 2.0437 |
| ENSMUSG00000050394 | Armcx6 | 0.0143 | 2.0394 |
| ENSMUSG00000035517 | Tdrd7 | 0.0000 | 2.0373 |
| ENSMUSG00000118640 | Gm7582 | 0.0094 | 2.0257 |
| ENSMUSG00000103739 | Gm37653 | 0.0291 | 2.0151 |
| ENSMUSG00000078894 | 2210418O10Rik | 0.0130 | -2.0076 |
| ENSMUSG00000025464 | Paox | 0.0153 | -2.0122 |
| ENSMUSG00000002108 | Nr1h3 | 0.0187 | -2.0710 |
| ENSMUSG00000053318 | Slamf8 | 0.0108 | -2.0876 |
| ENSMUSG00000039450 | Dcxr | 0.0259 | -2.0955 |
| ENSMUSG00000035863 | Palm | 0.0396 | -2.1067 |
| ENSMUSG00000014786 | Slc9a5 | 0.0210 | -2.1104 |
| ENSMUSG00000067714 | Lpar5 | 0.0497 | -2.1292 |
| ENSMUSG00000070369 | Itgad | 0.0319 | -2.1344 |
| ENSMUSG00000054619 | Mettl7a1 | 0.0415 | -2.1473 |
| ENSMUSG00000001027 | Scn4a | 0.0000 | -2.1568 |
| ENSMUSG00000030352 | Tspan9 | 0.0367 | -2.1614 |
| ENSMUSG00000112237 | D630033A02Rik | 0.0353 | -2.1644 |
| ENSMUSG00000030148 | Clec4a2 | 0.0106 | -2.1656 |
| ENSMUSG00000095285 | Ighv5-9 | 0.0420 | -2.1713 |
| ENSMUSG00000081205 | Gm5940 | 0.0092 | -2.1782 |
| ENSMUSG00000104063 | Pcdhgb7 | 0.0229 | -2.2149 |
| ENSMUSG00000096108 | Ighv11-2 | 0.0003 | -2.2156 |
| ENSMUSG00000120709 | Gm56535 | 0.0281 | -2.2415 |
| ENSMUSG00000066952 | Myo1h | 0.0224 | -2.2678 |
| ENSMUSG00000089998 | Phtf1os | 0.0007 | -2.2871 |
| ENSMUSG00000103293 | Gm5842 | 0.0328 | -2.2927 |
| ENSMUSG00000022428 | Cby1 | 0.0141 | -2.3010 |
| ENSMUSG00000004814 | Ccl24 | 0.0161 | -2.3176 |
| ENSMUSG00000024044 | Epb41l3 | 0.0122 | -2.3632 |
| ENSMUSG00000067321 | Gm7931 | 0.0157 | -2.3725 |
| ENSMUSG00000039115 | Itga9 | 0.0001 | -2.3761 |
| ENSMUSG00000054000 | Tusc1 | 0.0455 | -2.4336 |
| ENSMUSG00000045746 | B230317F23Rik | 0.0380 | -2.4386 |
| ENSMUSG00000090334 | Gm17149 | 0.0333 | -2.4522 |
| ENSMUSG00000028460 | Sit1 | 0.0155 | -2.5313 |
| ENSMUSG00000093483 | AA465934 | 0.0387 | -2.5327 |
| ENSMUSG00000086714 | 0610009E02Rik | 0.0220 | -2.5401 |
| ENSMUSG00000019966 | Kitl | 0.0030 | -2.5716 |
| ENSMUSG00000008540 | Mgst1 | 0.0306 | -2.5729 |
| ENSMUSG00000026712 | Mrc1 | 0.0079 | -2.5741 |
| ENSMUSG00000028517 | Plpp3 | 0.0214 | -2.5745 |
| ENSMUSG00000022218 | Tgm1 | 0.0107 | -2.5788 |
| ENSMUSG00000015854 | Cd5l | 0.0000 | -2.6258 |
| ENSMUSG00000116836 | Gm49727 | 0.0389 | -2.6735 |
| ENSMUSG00000097388 | Gm3200 | 0.0139 | -2.7634 |
| ENSMUSG00000094027 | Gm21762 | 0.0274 | -2.8723 |
| ENSMUSG00000111729 | Gm48038 | 0.0349 | -2.8945 |
| ENSMUSG00000120978 | Gm56588 | 0.0260 | -2.9240 |
| ENSMUSG00000015568 | Lpl | 0.0000 | -3.0250 |
| ENSMUSG00000074622 | Mafb | 0.0098 | -3.0942 |
| ENSMUSG00000042104 | Uggt2 | 0.0287 | -3.5125 |
| ENSMUSG00000025355 | Mmp19 | 0.0000 | -3.5242 |
| ENSMUSG00000049625 | Tifab | 0.0009 | -3.5741 |
| ENSMUSG00000064220 | H2ac18 | 0.0123 | -3.6208 |
| ENSMUSG00000049288 | Lix1l | 0.0007 | -3.6490 |
| ENSMUSG00000017390 | Aldoc | 0.0210 | -3.7208 |
| ENSMUSG00000072594 | Gm16439 | 0.0367 | -3.9336 |
| ENSMUSG00000076668 | Ighv7-4 | 0.0023 | -3.9364 |
| ENSMUSG00000036896 | C1qc | 0.0010 | -4.0449 |
| ENSMUSG00000052563 | D930048N14Rik | 0.0069 | -4.4024 |
| ENSMUSG00000089722 | Cd300ld5 | 0.0315 | -4.4456 |
| ENSMUSG00000036298 | Slc2a13 | 0.0098 | -4.5074 |
| ENSMUSG00000000440 | Pparg | 0.0010 | -4.7534 |
| ENSMUSG00000116872 | Gm6815 | 0.0202 | -4.8295 |
| ENSMUSG00000081201 | Smt3h2-ps4 | 0.0458 | -7.1253 |
| ENSMUSG00000090761 | Gm17201 | 0.0192 | -7.3749 |
| ENSMUSG00000036887 | C1qa | 0.0004 | -7.8317 |

“-” indicates downregulation of genes in the spirochetes with IsPDIA6 after *B. burgdorferi* stimulation.

**Table S3. The primers used in this study.**

| **Gene name** | **Primer sequence** |  |
| --- | --- | --- |
| Tick *actin* | F: GGCGACGTAGCAG R: GGTATCGTGCTCGACTC |  |
| Mouse *β-actin* | F: AGCGGGAAATCGTGCGTG  R: CAGGGTACATGGTGGTGCC |  |
| *Borrelia* *flaB* | F: TTCAATCAGGTAACGGCACA  R: GACGCRRGAGACCCTGAAAG |  |
| IsPDIA qPCR | F: GAGGGCGAGATGACCAAGTA R: AGCACGTCTTGCACAAATGA |  |
| IsPDIA4 qPCR | F: GCTCAAGGACTATGGCATCC R: TCTTGACCTCATGCACTTCG |  |
| IsPDIA5 qPCR | F: TCTCCGGTTACCCAACAGTC R: GGCTCCTTGGGATCTTTCAT |  |
| IsPDIA6 qPCR | F: CTCCAAGTACGGCATCAAGG R: GCCGCCATTGTACTCTTCAG |  |
| ds IsPDIA | F: TAATACGACTCACTATAGGGAGACGTTGGCTCAAGAAGAGGAC R: TAATACGACTCACTATAGGGAGATGCTCACAAAGAGCAGGTTG |  |
| ds IsPDIA6 | F: TAATACGACTCACTATAGGGAGAGGCGGAAAGAAAGACTCGT R: TAATACGACTCACTATAGGGAGAACTCGTTGATGCCCTCGTAG |  |
| ds GFP | F: TAATACGACTCACTATAGGGAGAGCGACGTAAACGGCCACAAGTT R: TAATACGACTCACTATAGGGAGACGGGTCTTGTAGTTGCCGTC |  |
| IsPDIA6-pEZT | F: AGGCGTTCAGTCTAGATACGGCCCTCACACTGAAGTCGTA  R: TTCAGACCGGCGGCCGCGAGCTCCACCCGCTTCTTGTCGG |  |
| IsPDIA6-pMT | F: CTCGCTCGGGAGATCTTACGGCCCTCACACTGAAGTC R: GCCCTCTAGACTCGAGGAGCTCCACCCGCTTCTTGTC |  |
| Trypsin Inhibitor | F: TTTTTCCTCGTTGGAACTGTC R: CACGGCATACTCCCTTGTC |  |
| Kunitz | F: CTCTGGCAGTGTGTTTCGTC  R: GGTACTCCTCCTCCAGCTCA |  |
| Trypsin Inhibitor | F: TAATACGACTCACTATAGGGAGACGTTTCTACTTCAACCAGTCG R: TAATACGACTCACTATAGGGAGACTTGTCCGTCACTTTCTTCG |  |
| Kunitz | F: TAATACGACTCACTATAGGGAGAGGATTTCGACCAGGGTTGT R: TAATACGACTCACTATAGGGAGAGCTGCGACCTGTAGTTGTTG |  |
| TNF-α qPCR | F: AGGCACTCCCCCAAAAGATG R: TGGTGGTTTGTGAGTGTGAGG |  |
|  |  |  |
| IL-18 qPCR | F: GACTCTTGCGTCAACTTCAAGG R: CAGGCTGTCTTTTGTCAACGA |  |
|  |  |  |
| IL-6 qPCR | F: ATACCACTCCCAACAGACCT R: CCAGTTTGGTAGCATCCATC |  |
|  |  |  |
| IL-1β qPCR | F: GCAGTGGTTCGAGGCCTAAT R: GCTGCTTCAGACACTTGCAC |  |
|  |  |  |
| CCL3 qPCR | F: GCCAGGTGTCATTTTCCTGAC R: CTCAAGCCCCTGCTCTACAC |  |
|  |  |  |
| IFN-γ qPCR | F: GAGGAACTGGCAAAAGGATGG R: ACCTGTGGGTTGTTGACCTC |  |
|  |  |  |
| CCL5 qPCR | F: GACAGCACATGCATCTCCCA R: GTGTCCGAGCCATATGGTGA |  |
|  |  |  |
| IL-10 qPCR | F: GTACAGCCGGGAAGACAATAAC R: GCATTAAGGAGTCGGTTAGCAG |  |
|  |  |  |
| TLR2 qPCR | F: AAGAGGAAGCCCAAGAAAGC R: AATGGGAATCCTGCTCACTG |  |
|  |  |  |
| IL-4 qPCR | F: CGGATGCGACAAAAATCAC R: CGTTTGGCACATCCATCTC |  |
|  |  |  |
| IL-12 qPCR | F: ATCGTTTTGCTGGTGTCTCC R: CTTCTTCAGGCGTGTCACAG |  |
|  |  |  |
| TGF-β qPCR | F: TGGAGCAACATGTGGAACTC R: TGCCGTACAACTCCAGTGAC |  |
|  |  |  |
| IL-17 qPCR | F: TCATCTGTGTCTCTGATGCTGTTG R: TCGCTGCCTTCACTGT |  |
|  |  |  |
| IsPDIA6-M1 | F: TGCTCCCTGGagcGGACACagcC R: AAGAACTCGACGATCCAC |  |
| IsPDIA6-M2 | F: TGCACCCTGGagcGGCCACagcA R: AAGAACTCCACCAGCCAGAGGTCC |  |
